# Interleukin-1 signaling and CD4^+^ T cells control B cell recruitment to the lungs in chronic beryllium disease

**DOI:** 10.3389/fimmu.2025.1479348

**Published:** 2025-01-28

**Authors:** Joseph M. Gaballa, Caley Valdez, Douglas G. Mack, Faiz Minhajuddin, Masoom Raza, Tabrez A. Mohammad, Allison K. Martin, Andrew Getahun, Charles A. Dinarello, Andrew P. Fontenot, Shaikh M. Atif

**Affiliations:** ^1^ Department of Medicine, University of Colorado Anschutz Medical Campus, Aurora, CO, United States; ^2^ Greehey Children's Cancer Research Institute, The University of Texas Health Science Center at San Antonio, San Antonio, TX, United States; ^3^ Department of Immunology and Microbiology, University of Colorado Anschutz Medical Campus, Aurora, CO, United States

**Keywords:** interleukins, IL-1, IL-1Ra, Anakinra, chemokines, inflammation, beryllium

## Abstract

Chronic beryllium disease (CBD) is a debilitating pulmonary disorder that occurs due to persistent exposure to beryllium (Be) particles in the workplace. Be-exposure causes activation of the innate immune system, resulting in the secretion of interleukins and chemokines that drive the accumulation of B and T cells in the lungs. However, the mechanisms by which innate molecules influence the recruitment of B cells and B cell-mediated protection in CBD are poorly understood. In this study, we employed multiple approaches to examine the role of innate immune signaling and CD4^+^ T cells in B cell recruitment and function in the lungs. We show that the absence or blocking of IL-1R1 signaling prevents the recruitment of B cells to the lungs of BeO-exposed mice. Additionally, we show that B cell recruitment to the lungs depends on the chemokine receptor, CXCR5, and CD4^+^ T cells. In BeO-exposed mice, lung B cells down-regulate IgM but showed an increased IgD and CD44 surface expression. Further, RNA sequencing of pulmonary tissue-specific B cells in CBD revealed distinct gene signatures compared to splenic B cells, with increased expression of pathways involved in antigen presentation, tight junction interactions, and interferon signaling. Overall, our study shows that B cell recruitment and aggregate formation during CBD depend on sequential activation of innate and adaptive immune responses.

## Introduction

Chronic beryllium disease (CBD) is an immune-mediated granulomatous lung disorder caused by exposure to beryllium in the environment (1). CBD most commonly presents in individuals working in manufacturing industries where they are passively exposed to high levels of beryllium in the workplace (2, 3). CBD is strongly associated with a specific HLA-allele, *HLA-DPB1*, which contains a negatively charged glutamic acid at position 69 of the β-chain (4, 5). Individuals expressing this allele are predisposed to an increased risk of disease progression (6). The pathogenesis of CBD involves binding of positively charged beryllium cations to negatively charged glutamic acid residues in the HLA molecules, thus creating neo-antigens, which induce a robust T cell response (7, 8). Clinical manifestations of CBD include cough, fever, and night sweats, similar to other granulomatous diseases of the lung such as tuberculosis and sarcoidosis (9).

Activation of immune cells by metals, foreign antigens, and infectious pathogens can lead to inflammation characterized by the release of cytokines, chemokines, and cellular DNA (10–12). These cellular products influence the function and phenotype of immune cells and drive the recruitment of additional immune cells to sites of inflammation. In the context of CBD, previous studies have shown that the uptake of beryllium particles results in the activation and death of resident alveolar macrophages, leading to the release of damage-associated molecular patterns. These molecules then initiate inflammatory cascades in the lungs that lead to the recruitment of B cells and IFNγ producing T cells, causing lung scarring, which is a hallmark of CBD (13, 14).

The interleukin-1 (IL-1) family of cytokines and receptors includes 22 members, mainly produced by cells of the innate and adaptive immune systems, and play broad roles in the function and recruitment of immune cells (15, 16). IL-1 signaling promotes granuloma formation, which helps sequester infectious and non-infectious particulate matter. IL-1α and IL-1β are the initial members of the IL-1 family that utilize interleukin-1 receptors (IL-1R) to initiate a signaling cascade leading to the secretion of cytokines and chemokines. The absence of IL-1α or IL-1R signaling causes impaired granuloma formation (17, 18). Chemokines are another class of secreted molecules that are crucial for driving immune cell recruitment to sites of inflammation (19). Important chemokines that are released early in the lungs after beryllium exposure are CXCL13, CCL19, and CCL21 (20, 21), however, the role that these chemokines play in recruiting immune cells to the lungs during CBD remains unclear.

The magnitude of the pathogenic response to beryllium exposure is dependent on CD4^+^ T cells, where initially CD4^+^ T cells recognize beryllium neoantigens on the MHC class II molecules of antigen-presenting cells (22). This priming of CD4^+^ T cells allows them to become activated, characterized by the increased surface expression of CD44, CD69, downregulation of L-selectin CD62L, and the secretion of proinflammatory cytokines such as IFNγ, TNF-α, and IL-2 which are necessary for maintaining an inflammatory state in the lungs (21). Contrary to the role of CD4^+^ T cells in CBD, B cells have been shown to play a protective role in murine models of CBD, with B cells representing a significant portion of infiltrating leukocytes, and depletion of B cells leading to worse disease outcomes in mice (20). Thus, understanding the mechanisms that drive B cell infiltration during CBD is crucial for elucidating their protective role during disease and may offer a novel treatment approach for human disease.

Therefore, in the present study, we sought to identify mechanisms that drive B cell recruitment to the lungs during CBD. We found that IL-1 family members, specifically IL-1α and IL-1R1, are key drivers of B cell recruitment to the lung. Further, we found that CD4^+^ T cell recruitment to the lung precedes the recruitment of B cells and show that CD4^+^ T cells are indispensable for recruiting B cells to the lung. The recruitment of B cells to the lungs was also found to be dependent on the chemokine receptor, CXCR5. Transcriptional analysis of pulmonary B cells during CBD revealed a distinct gene signature compared to splenic B cells, with increased expression of genes involved in pathways associated with antigen presentation, tight junction interactions, and interferon signaling. Taken together, our work shows that CD4^+^ T cells and IL-1 signaling are required for B cell recruitment to the lung through a CXCR5-CXCL13-dependent process.

## Methods

### Mice

C57BL/6J, B6.129S7-Il1r1tm1Imx/J, and B6.129S2(Cg)-*Cxcr5^tm1Lipp^*/J mice were purchased from Jackson Laboratory. Previously described HLA-DP2 Tg FVB/N and C57BL/6J mice were generated and maintained in our mouse colony (21, 23). Non-HLA-DP2 strains were used to assess the role of innate molecules in the recruitment of adaptive immune cells, whereas HLA-DP2 Tg strains were used to examine the role of adaptive immune cells in CBD. All experiments were approved by the Institutional Animal Care and Use Committee of the University of Colorado Denver, following the National Institutes of Health guidelines for using live animals. The University of Colorado Denver is accredited by the American Association for Accreditation of Laboratory Animal Care.

### Exposure of mice to beryllium oxide

Six to eight-week-old mice were exposed to 50 µL of PBS-containing 100 μg of endotoxin-free BeO (NIST, standard reference material 1877) (24, 25) or PBS via oropharyngeal aspiration as previously described (21). The endotoxin level was less than 20 μg in 50 mL of BeO preparations as examined by a Limulus amebocyte assay (Sigma-Aldrich). Mice were briefly anesthetized with isoflurane to facilitate aspiration. In our murine model of CBD, all mice were sensitized via oropharyngeal aspiration with three doses of BeO or PBS (GIBCO) on days 0,1 and 2 and then sacrificed on day 12, or they were boosted with either BeO or PBS on days 14, 15, 18, and 19, and then sacrificed on day 21.

### Antibody treatment

A monoclonal anti-mouse CD20 antibody (clone 5D2) and a murine IgG2a isotype control antibody provided by Genentech were used as described previously (21). For blocking IL-1α and IL-1R1, mice were injected intraperitoneally with 400 μg of an anti-IL-1α monoclonal antibody (clone FLO1-2A, Biotech, USA, Inc., Austin, TX) or Anakinra (recombinant IL-1 receptor antagonist; IL-1Ra), respectively, at days -1, 1, 3, 6, and 9. CD4^+^ T cells were depleted using 100 μg of an anti-CD4 monoclonal antibody (clone GK1.5, BioXcell, USA) or isotype control antibody. Intraperitoneal injection of antibodies was administered on either day -1, 4, 9, or 15. B cell recruitment was analyzed in the bronchoalveolar lavage (BAL) and lungs of BeO-exposed mice.

### B cell adoptive transfer

B cells were purified from the spleens of 6–8-week-old C57BL/6 WT mice using a Milteyni Biotec mouse B cell isolation kit (Milteyni Biotech, USA). Single-cell suspensions were prepared, and RBC lysis was performed using ACK lysis buffer (BD Biosciences, USA) before adding microbeads. Enriched B cells were collected in PBS, and the purity was confirmed to be greater than 95% via flow cytometry. 1x10^7^ cells were transferred via tail vein injection into the recipient mice at days -1, 8, and 15.

### Preparation of single-cell suspensions of splenocytes and lung cells

CD45-PE or APC-Cy7 monoclonal antibodies (mAbs, clone 30-F11) (5 μg/mouse) were injected intravenously via the retro-orbital route 2 minutes before sacrifice to discriminate between tissue-localized and circulating cells (21, 26–30). Mice were euthanized under anesthesia and the lungs were perfused with ice-cold PBS. Lungs were removed, minced, and digested in complete RPMI containing 1mg/ml collagenase (Sigma-Aldrich). After 30 minutes, collagenase-digested lungs were sequentially disrupted with 16G and 18G needles. Collagenase was quenched with cold MACS buffer (1X PBS and 2% FBS), and lung cells were centrifuged at 1,500 rpm for 5 minutes.

The tissue was pressed through a 100 µm cell strainer to obtain single cells from the spleen. Erythrocytes were lysed with ACK lysis buffer, and lung cells were filtered through a 70 µm cell strainer and re-suspended in complete RPMI (RPMI-C), consisting of RPMI 1640 (HyClone) supplemented with 10% heat-inactivated FBS (HyClone) and penicillin and streptomycin (Invitrogen).

### Isolation of BAL cells and BALF

BAL was collected after perfusing the lungs with 10 mL of ice-cold PBS (GIBCO) and accomplished by tracheal cannulation. For bronchoalveolar lavage fluid (BALF) collection, 1 mL of PBS was instilled, which yielded a 0.75 mL return volume. BALF was centrifuged at 2,000 g for 2 minutes, and the acellular fraction was frozen at -80° C for analysis of lung injury. The cell pellets obtained after centrifugation were then used for immune cell assays.

### Lung injury and cytokine analysis

Protein leak, a marker of lung injury, was quantified by measuring protein concentrations in the BALF using the bicinchoninic acid (BCA) assay (ThermoScientific). Cytokine levels were measured from the frozen BALF samples using indirect ELISA kits (R&D Systems). Absorbance readings were measured at 570 and 450 nm using a VMax microplate reader (Molecular Devices). Concentrations were calculated using GraphPad Prism (version 10.1.1).

### Flow cytometry

Fluorescently labeled monoclonal antibodies were diluted in PAB buffer (PBS containing 1% FCS, 0.05% sodium azide, and 0.5 µg/ml CD16/CD32 (Tonbo; 2.4G2). Cells were incubated for 30 minutes at 4° C and then washed and re-suspended in PBS. The following antibodies (Vender; clone) were used for multi-parameter FACS analysis: CD3 (Tonbo; 145-2C-11), CD4 (BioLegend; RM4-5), CD8 (BioLegend; 53-6.7), CD19 (BioLegend; 6D5), B220 (BioLegend, RA3-682), CD11b (BioLegend, M1/70), IA/IE (BioLegend, M5.114.15.2), and Ly6G (BioLegend, 1A8), IgM (BioLegend, RMM1), IgD (BioLegend, 11-26c.2a), CD44 (BioLegend, IM7), CD86 (BioLegend, GL-1), CD69 (BioLegend, H1.2F3). Data was acquired using a BD FACS CANTO II flow cytometer (BD USA), and all post-acquisition analyses were performed in FlowJo (v10.06).

### Lung histology

Lungs were inflated and stored in 10% neutral-buffered formalin for 24 hours and transferred to 70% ethanol for histopathologic analysis. Immunohistochemistry on paraffin-embedded sections of lung slides was performed by Histowiz. Briefly, each section was stained with Hematoxylin and eosin, and for B cell staining, a 1:10000 dilution of rabbit anti-B220 (Novus Biologicals, RA3-6B2) was applied using a Bond RX automated stainer (Leica Biosystems). Primary antibodies were detected using a rabbit-specific IHC polymer detection kit (Abcam) and the antibody-bound antigen sites were visualized using 3,3′-Diaminobenzidine (DAB) staining. Separate lung sections were stained with hematoxylin and eosin (H&E), and whole slide scanning was performed on an Aperio AT2 (Leica Biosystems). The tiff files were analyzed using QuPath, v.0.5.1 software. Briefly, stain vector values were automatically determined by the software, and cellular aggregates were counted by adjusting the cell detection threshold, maximizing the difference between areas containing perivascular mononuclear infiltrates and unaffected areas (background).

### Library construction and RNA sequencing

RNA sequencing was performed on bead-purified and FACS-sorted lung and spleen B cells. Library construction and sequencing were performed by the Genomics and Microarray Core Facility at the University of Colorado Anschutz Medical Campus. RNA quality and integrity were first assessed using an Agilent Tape Station 2200. The library was constructed using the Illumina TruSeq mRNA library construction kit. Paired-end sequencing was performed using an Illumina HiSeq 4000 for 125 cycles. Sequencing data was processed using the Illumina BaseSpace online platform and assessed using FASTQC. The FASTQ Toolkit was used to filter, adaptor trim and quality check trim reads. Reads were aligned to UCSC mm9 genome build using TopHat and read counts (gene expression levels) were obtained using HTSeq. Differential expression analysis was performed using DESeq, and the following criteria determined up and down-regulated genes: (i) fold change > 2, (ii) average RPKM (reads per kilobase per million mapped reads) > 1, and (iii) false discovery rate < 0.05. Gene Ontology (GO)-pathway analysis was performed using DAVID (31).

### Statistics

All statistical tests were performed in GraphPad Prism (v.10.1.1) using unpaired Student’s *t*-test or One-way ANOVA. P values <0.05 were deemed statistically significant.

## Results

### IL-1R1 signaling controls BeO-induced B cell recruitment to the lungs

**Figure 1:**
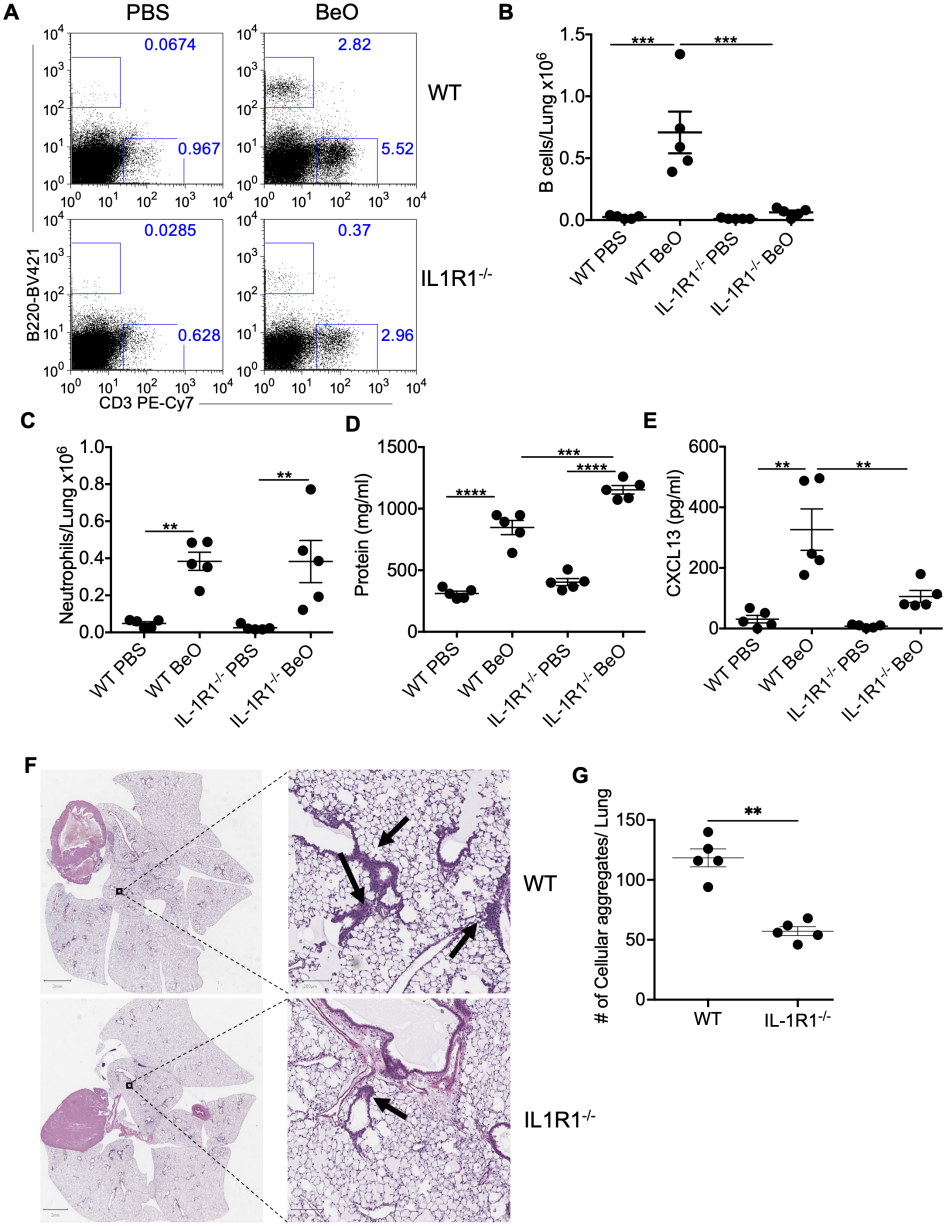
IL-1R1 signaling controls BeO-induced B cell recruitment to the lungs. Wildtype (WT) and interleukin-1 receptor 1 knockout (IL-1R1^-/-^) mice were exposed to BeO and sacrificed on day 21. **(A)** Flow cytometry plots show the recruitment of adaptive immune cells in the lungs of PBS or BeO-exposed mice. Graph plots show a total number of tissue-specific B220+ B cells **(B)** and neutrophils **(C)** in the lungs of PBS and BeO exposed WT and IL-1R1^-/-^ mice on day 21. **(D)** Total protein concentration in the BALF measured by BCA, and **(E)** CXCL13 levels in the BALF on day 21 measured by ELISA. **(F)** Hematoxylin and eosin (H&E) staining of BeO-exposed lung tissues from WT (top) and IL-1R1^-/-^ (bottom) mice. **(G)** Cellular aggregates in the lungs of WT and IL-1R1^-/-^ mice exposed to BeO were quantified using QuPath. Data are representative of three independent experiments having n=5 mice per group. One-way ANOVA was used to test statistical differences among the groups. P<0.05 (*) is considered statistically significant. P < 0.01 (**), P < 0.001 (***), P < 0.0001 (****).

Previously, we and others showed that exposure to silica or beryllium particles induces the secretion of both IL-1α and IL-1β in the BAL (13, 20, 32). Here, we investigated the role of interleukin-1 signaling (IL-1α or 1β) in the recruitment of B cells to the lungs of BeO-exposed mice. We used IL-1R1 KO (IL-1R1^-/-^) mice to study the contribution of IL-1R1 signaling in driving B cell responses. We first examined the frequency of B cells in the lungs of BeO-treated WT and IL-1R1^-/-^ mice (**Figure 1A**) using the gating strategy described in **Supplementary Figure 1**. At day 21, an increase in the absolute number of B cells in the lung of BeO-treated WT mice compared to IL-1R1^-/-^ mice was seen (**Figure 1B**). We also examined B cells in the spleen of PBS and BeO-treated WT and IL-1R1^-/-^ mice. The frequencies of B cells were similar in these mice, suggesting that the decrease in B cells in the lungs of treated mice was independent of B cell frequencies in the spleen (**Supplementary Figure 2**). In the absence of IL-R1 signaling, we also noticed decreased CD3^+^ T cell recruitment (**Figure 1A**). The lower frequency of T cells in the lungs of IL-1R1^-/-^ mice might be due to the loss of IL-1 signaling impacting soluble mediators required for their migration and recruitment. Next, we compared BeO-induced lung inflammation in BeO-treated WT and IL-1R1^-/-^ mice. Increased neutrophil or protein presence are markers of inflammation and injury (21). We measured the neutrophils in BeO-exposed mice, and both WT and IL-1R1^-/-^ mice showed a similar number of neutrophils in the BAL collected at day 21 (**Figure 1C**). However, in contrast to the similar neutrophil response, we observed a significant increase in the protein leakage into the BALF (**Figure 1D**), suggesting an enhanced lung injury in IL-1R1^-/-^ mice. We have also examined CXCL13, a B cell homeostatic chemokine that is critical for the recruitment of B cells to the lung in response to BeO in the BALF of BeO-treated groups. CXCL13 was significantly diminished in BeO-exposed IL-1R1^-/-^ mice compared to WT mice (**Figure 1E**). The reduced levels of CXCL13 in IL-1R1^-/-^ mice compared to WT mice are likely due to decreased IL-1/IL-1R1 signaling from innate immune cells and stromal cells that ultimately drive the production of CXCL13 and the recruitment of B cells. Our previous study demonstrated that cellular aggregates in the lungs of mice exposed to BeO contain substantial populations of B cells (20). Here, we show a significant reduction in the number of lymphoid aggregates in the lungs of BeO-exposed IL-1R1^-/-^ mice (**Figures 1F, G**). Taken together, these findings suggest an important role of IL-1R1 signaling in driving BeO-induced B cell responses.

### Neutralization of IL-1α and IL-1R1 abrogates BeO-induced lung inflammation and B cell recruitment

**Figure 2:**
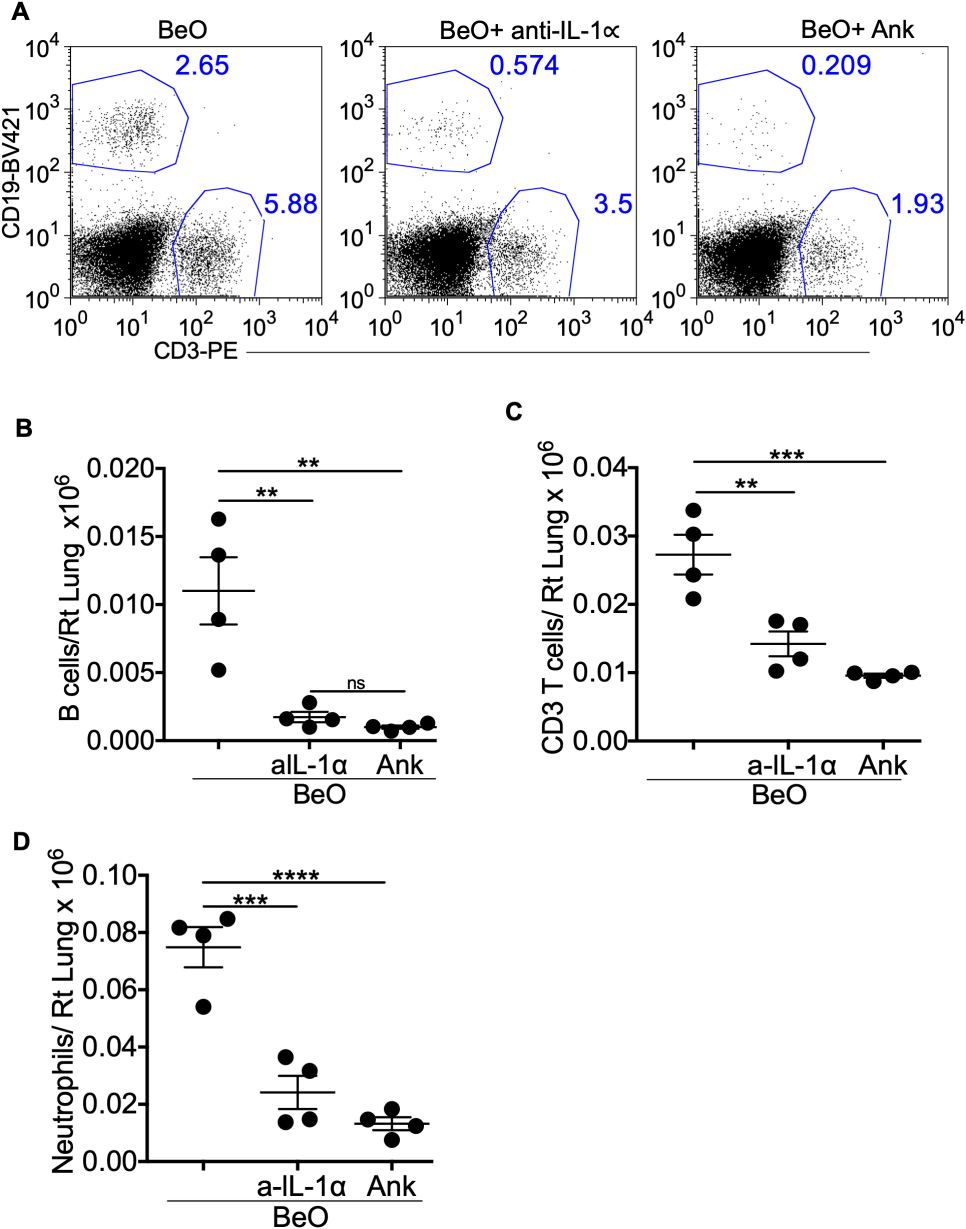
Neutralization of IL-1α and IL-1R1 abrogates BeO-induced lung inflammation and B cell recruitment. BeO exposed mice were treated with an isotype control antibody, anti-IL-1α, or Anakinra, and sacrificed on day 12. **(A)** Flow cytometry analysis of BALF shows CD19^+^ B cell and CD3^+^ T cell populations on day 12. **(B)** Total number of tissue-specific B220^+^ B cells in the lungs of antibody-treated mice on day 12. **(C)** Total number of CD3^+^ T cells in the lungs of antibody-treated mice on day 12. **(D)** Total number of neutrophils in the lungs on day 12. Data are representative of three independent experiments, 4 mice per group. One-way ANOVA was used to test statistical differences among the groups. P<0.05 (*) is considered statistically significant. P < 0.01 (**), P < 0.001 (***), P < 0.0001 (****).

To establish the role of IL-1 signaling pathways in driving BeO-induced B cell recruitment to the lungs, we used either a neutralizing antibody against IL-1α or a recombinant version of IL-1 receptor antagonist (IL-1Ra), Anakinra, to block the IL-1/IL-1R1 signaling pathway. Previously, we showed that 12 days post sensitization with three doses of BeO was adequate to recruit adaptive immune cells and form cellular aggregates in the lungs (21). Additionally, considering the possibility of an immune response against the neutralizing antibodies, we decided to test the efficacy of these antibodies on the recruitment of immune cells on day 12 rather than day 21 post-sensitization with BeO. Mice treated with anti-IL-1α or Anakinra showed a greater than 5-fold reduction in the frequency and number of tissue-specific B cells in the lungs on day 12 (**Figures 2A, B**). Additionally, we noticed a significant impact of these treatments on the recruitment of total T cells in the lungs (**Figure 2C**). Next, we examined the effect of anti-IL-1α or Anakinra in preventing BeO-induced inflammation in the lungs. On day 12, we found that blocking of IL-1α or IL-1R resulted in reduced inflammation in the lungs of mice sensitized with BeO as suggested by a 4-fold decrease in neutrophils (**Figure 2D**).

### Adoptively transferred B cells protect against BeO-induced lung injury

**Figure 3:**
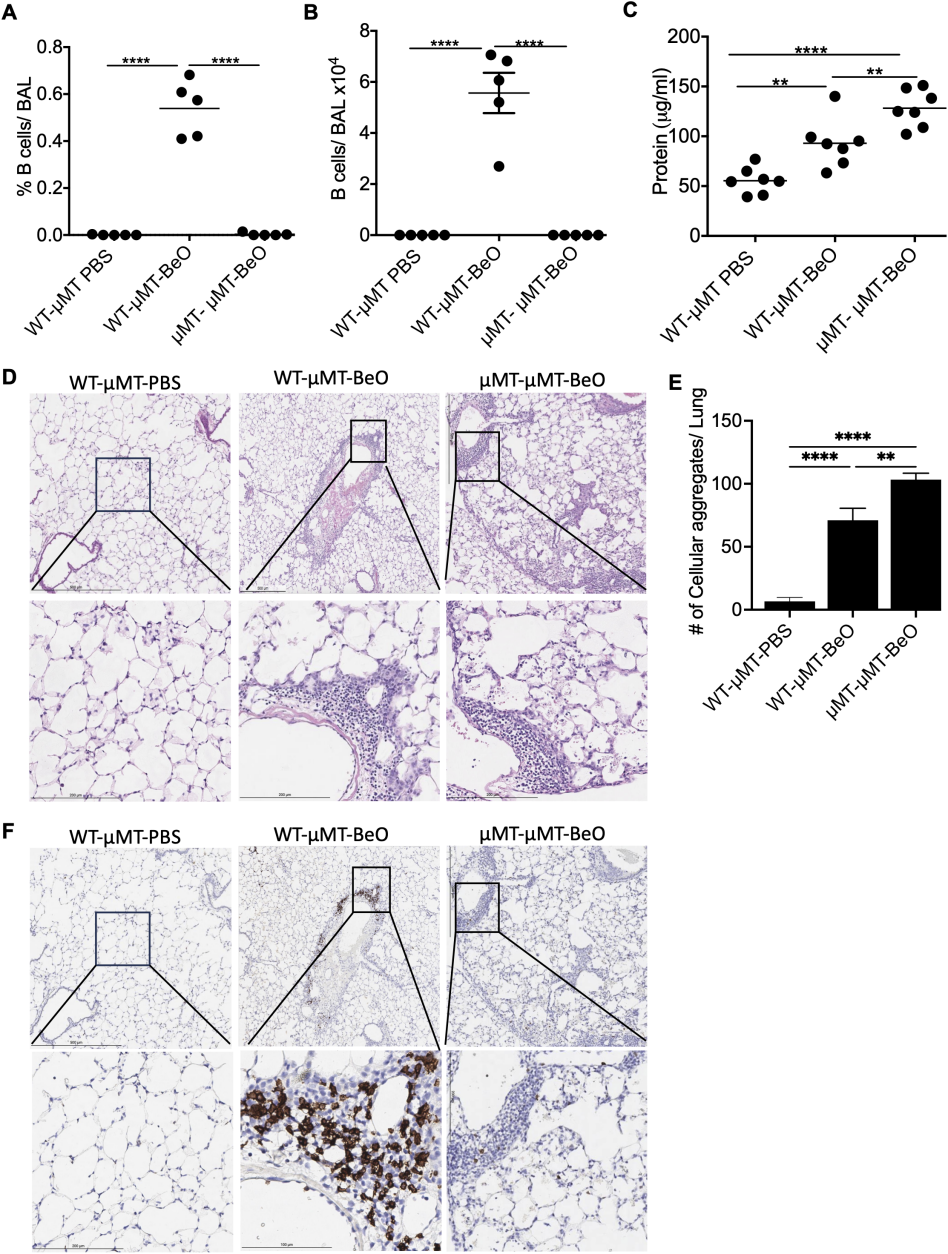
Adoptively transferred B cells protect against BeO-induced lung injury. μMT mice were adoptively transferred with wild-type B cells purified from the spleen or with splenocytes from μMT mice. Recipient mice were sensitized and boosted with BeO using the previously described protocol. Percent frequency **(A)** and total number of B cells **(B)** in the BAL of BeO-exposed mice as examined on day 21. **(C)** Protein leak in the bronchoalveolar lavage fluid (BALF) of μMT mice adoptively transferred with WT splenic B cells or μMT splenocytes and exposed to BeO on day 21. **(D)** Hematoxylin and eosin staining show low magnification (top) and high magnification (bottom), and **(E)** cellular aggregates quantified in the lungs of mice adoptively transferred with WT splenic B cells or splenocytes from μMT mice into μMT mice and exposed to BeO. Cellular aggregates were quantified using QuPath. **(F)** Immunohistochemical (IHC) staining of B cells low magnification (top) and high magnification (bottom) on day 21 using an anti-rabbit B cell antibody in the lungs of mice adoptively transferred with WT splenic B cells or splenocytes from μMT mice into μMT mice and exposed to BeO. Data are representative of three independent experiments, 3-5 mice per group. One-way ANOVA was used to test statistical differences among the groups. P<0.05 (*) is considered statistically significant. P < 0.01 (**), P <l 0.0001 (****).

Beryllium exposure induces innate immune responses that participate in the production of CXCL13, a B cell homeostatic chemokine. Thus, we have examined the recruitment of B cells to the lungs of WT and μMT mice exposed to BeO. μMT mice lack mature B cells and are defined as B cell-deficient mice. Flow cytometry analysis confirmed the absence of B cells in the lungs of μMT mice, while B cells were recruited normally to the lungs of BeO exposed WT mice (**Supplementary Figures 3A, B**). To study how B cells mediate protection against BeO-induced lung injury in Be-exposed mice, we utilized an adoptive transfer approach. Naïve B cells (1x10^7^) enriched from the spleens of wildtype mice were transferred into B cell-deficient (μMT) mice and treated with PBS (WT-μMT-PBS) or BeO (WT-μMT-BeO). In the experimental control group, B cell-deficient mice (μMT-μMT-BeO) were adoptively transferred with splenocytes (1x10^7^) from B cell-deficient mice. On day 21, we observed B cells in the alveolar space and peribronchovascular region of the lungs of BeO-treated mice adoptively transferred with B cells, suggesting that adoptively transferred B cells migrated to the lungs in response to BeO exposure (**Figures 3A, B**). Additionally, significantly fewer B cells were detected in the lungs of the μMT-μMT-BeO-treated group as the splenocytes transferred from the μMT mice lacked mature B cells. We measured protein leakage in the BALF of mice adoptively transferred with WT B cells or μMT splenocytes. The WT-μMT-BeO group showed reduced levels of protein in the BALF as compared to the μMT -μMT-BeO-treated group (**Figure 3C**). H&E staining showed an increased presence of cellular aggregates in the lungs of μMT-μMT-BeO mice compared to the WT-μMT-BeO mice (**Figures 3D, E**). Next, we used immunohistochemistry to track B cell recruitment to the lungs of PBS or BeO-exposed mice. The adoptively transferred B220^+^ B cells were localized within the peribronchovascular space in WT-μMT-BeO treated mice (**Figure 3F**), whereas B cell staining was absent in the lungs of μMT-μMT-BeO mice (**Figure 3F**). Overall, this data suggests that adoptively transferred B cells are seeded in the lungs and protect the lungs from BeO-induced inflammation.

### CXCR5 expression is required for B cell recruitment to the lung in response to BeO exposure

**Figure 4:**
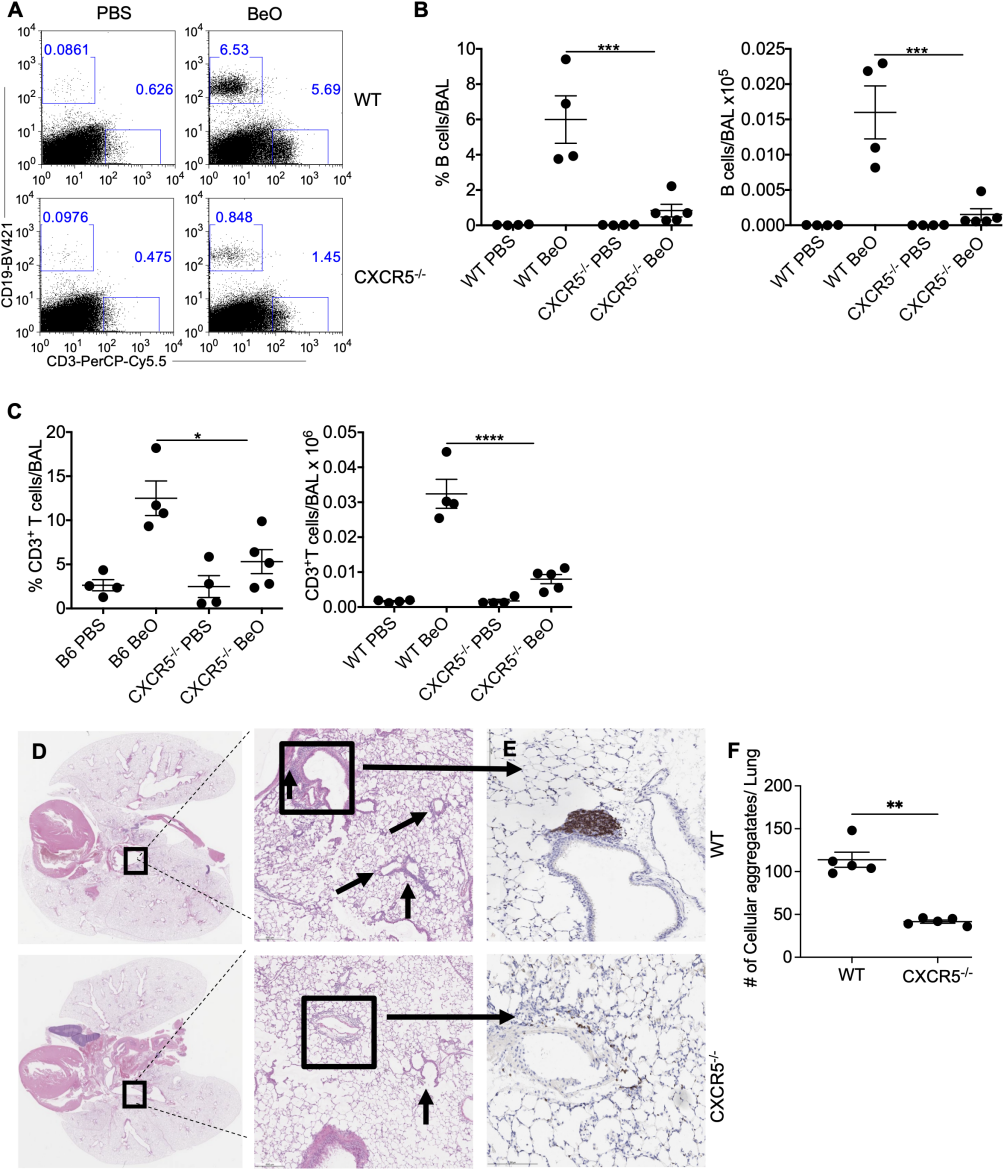
CXCR5 expression is required for B cell recruitment to the lung in response to BeO exposure. WT and CXCR5^-/-^ mice were sensitized with seven doses of BeO (100 μg) on days 0, 1, 2, 14, 15, 18, and 19 and sacrificed on day 21. **(A)** Representative dot plot shows the presence of CD19^+^ and CD3^+^ T cells and **(B)** cumulative percent frequency (left) and total number (right) of B cells in the BAL of BeO-exposed mice. **(C)** Percent frequency (left) and the total number of CD3^+^ T cells (right) in the BAL of BeO-exposed WT and CXCR5^-/-^ mice on day 21. **(D)** Hematoxylin and eosin staining and **(E)** immunohistochemical (IHC) staining of B cells using an anti-rabbit B cell antibody of BeO-exposed lung tissues. **(F)** Cellular aggregates in the lungs of WT and CXCR5^-/-^ mice were quantified using QuPath. Data are representative of three independent experiments, 4-5 mice per group. One-way ANOVA was used to test statistical differences among the groups. P<0.05 (*) is considered statistically significant. P < 0.05 (*), P < 0.01 (**), P < 0.001 (***), P < 0.0001 (****).

The CXCL13/CXCR5 signaling axis plays an important role in the maturation, activation, and recruitment of B cells in the secondary lymphoid organs (33). In our murine model of CBD, BeO treatment leads to increased expression of CXCL13 in the lungs (20). To study the involvement of CXCR5 in mediating B cell recruitment following BeO exposure, we treated WT and CXCR5-deficient (CXCR5^-/-^) mice with PBS or BeO. WT and CXCR5^-/-^ mice had similar numbers of B cells in the spleen at baseline upon treatment with PBS or BeO (**Supplementary Figures 4A, B**). BeO exposure resulted in a significant increase in the recruitment of lymphocytes to the lungs (**Figure 4A**). The frequency and number of B cells in the BAL of WT mice were significantly higher compared to the CXCR5^-/-^ mice (**Figure 4B**). Additionally, we observed an effect of CXCR5 deficiency on the recruitment of CD3^+^ T cells to the lungs. Mice lacking CXCR5 show a 2.5-fold reduction in the frequency of CD3^+^ T cells (**Figure 4C**, left) and a 3-fold reduction in the number of CD3^+^ T cells (**Figure 4C**, right) in the BAL. H&E staining of lung tissues revealed an increased presence of cellular aggregates in the WT-treated mice compared to CXCR5^-/-^ mice (**Figure 4D**). The presence of a large number of aggregates correlated with the increased accumulation of B cells in WT BeO-exposed lungs, whereas B cells were nearly absent in the lungs of CXCR5^-/-^ mice (**Figures 4E, F**). Overall, the data indicates that the CXCL13/CXCR5-signaling axis is crucial in recruiting adaptive immune cells, including B cells, and the formation of cellular aggregates.

### Kinetics of leukocyte recruitment to the lung in response to BeO exposure

**Figure 5:**
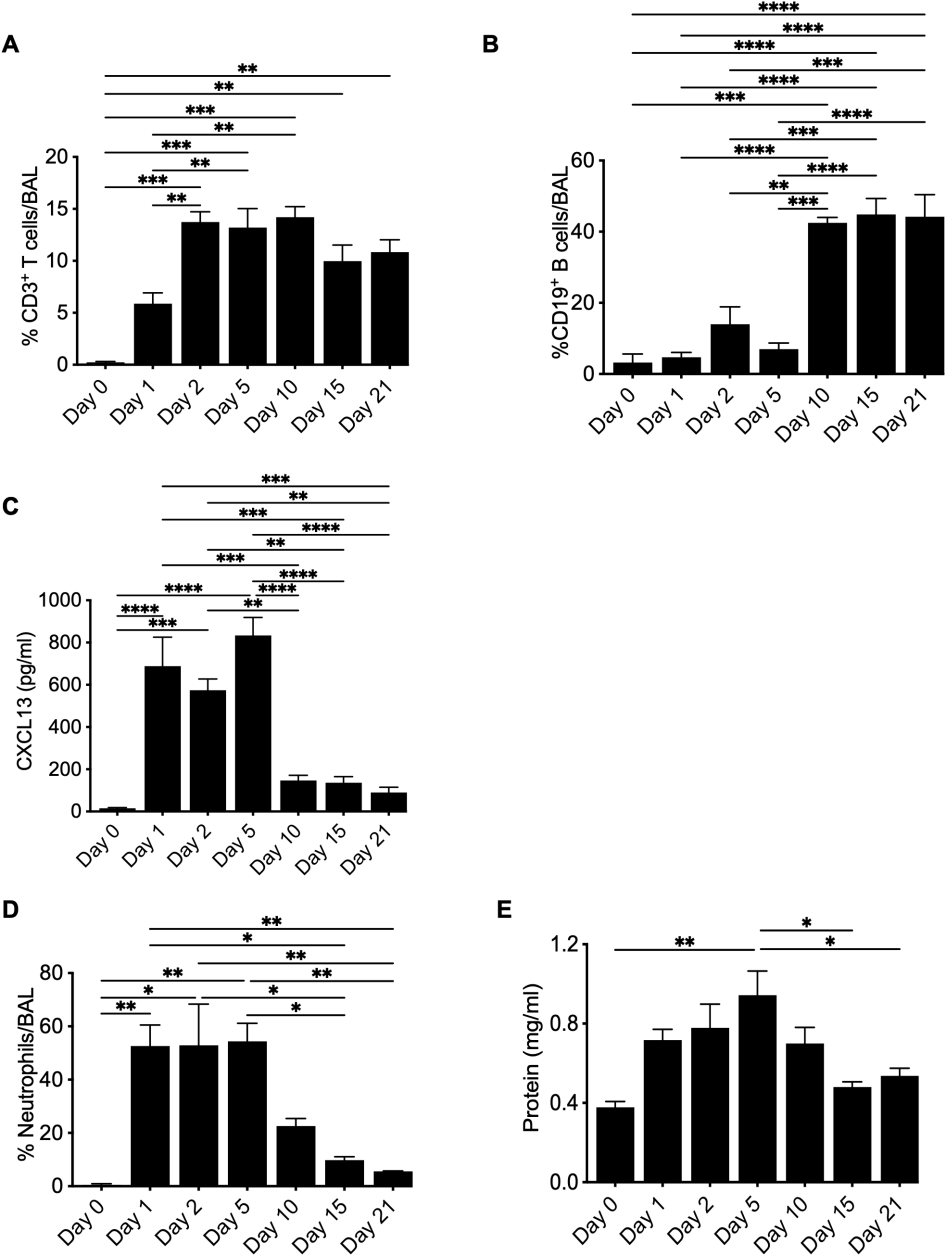
Kinetics of leukocyte recruitment to the lung in response to BeO exposure. HLA-DP2 FVB/N Tg mice were sensitized with three doses of BeO (100 μg) on days 0, 1, and 2. Mice (3-4) per time point were sacrificed, and immune cells were analyzed by flow cytometry. Graph plots show the percent frequency of **(A)** CD3^+^ T cells, **(B)** CD19^+^ B cells, and **(C)** CXCL13 in the bronchoalveolar lavage fluid. Graph plots show the percent frequency of neutrophils **(D)** and protein leak **(E)** in the bronchoalveolar lavage fluid (BALF) of BeO exposed mice. Data are representative of two independent experiments, 3-4 mice per group. One-way ANOVA was used to test statistical differences among the groups. P<0.05 (*) is considered statistically significant. P < 0.05 (*), P < 0.01 (**), P < 0.001 (***), P < 0.0001 (****).

In HLA-DP2 Tg FVB/N mice, BeO exposure results in the infiltration of leukocytes, the formation of cellular aggregates, and granuloma formation (20). Previously, we evaluated adaptive immune responses in a chronic model of beryllium disease that requires seven exposures to BeO (100 μg) at days 0, 1, 2, 14, 15, 18, and 19, with results evaluated at day 21. However, to understand the coordinated role of immune cells in disease establishment and progression, we evaluated the response after three doses of BeO (100 μg) administered at days 0, 1, and 2 (i.e., sensitization phase). We tracked the recruitment of CD3^+^ T cells and B cells in the alveolar space by flow cytometry. T cell recruitment occurred early and remained elevated for the duration of the experiment (**Figure 5A**). Conversely, the frequency of B cells was not increased until day 10 but remained elevated until day 21 (**Figure 5B**). Early recruitment of CD3^+^ T cells suggests that T cells precede B cell recruitment and could have a role in driving B cell recruitment. Furthermore, CXCL13 expression in the BALF was detected at days 1-5 and diminished during the progression phase of the disease (**Figure 5C**), suggesting an early chemokine gradient after BeO exposure that is sufficient for the recruitment of B cells. However, the data also suggests that B cell aggregates do not require CXCL13 for their maintenance after the establishment of the disease. Next, we quantified BeO-induced lung injury by measuring the presence of neutrophils and protein leak in the BALF. Neutrophils arrived early but then gradually dissipated as time progressed, in contrast to the kinetics of B cells (**Figure 5D**). A similar phenomenon was seen with protein leak decreasing upon arrival of the B cells (**Figure 5E**), suggesting that Be-induced inflammatory responses occur early after exposure and wane with time.

### B cells are dispensable for CD4^+^ T cell recruitment and CXCL13 expression

**Figure 6:**
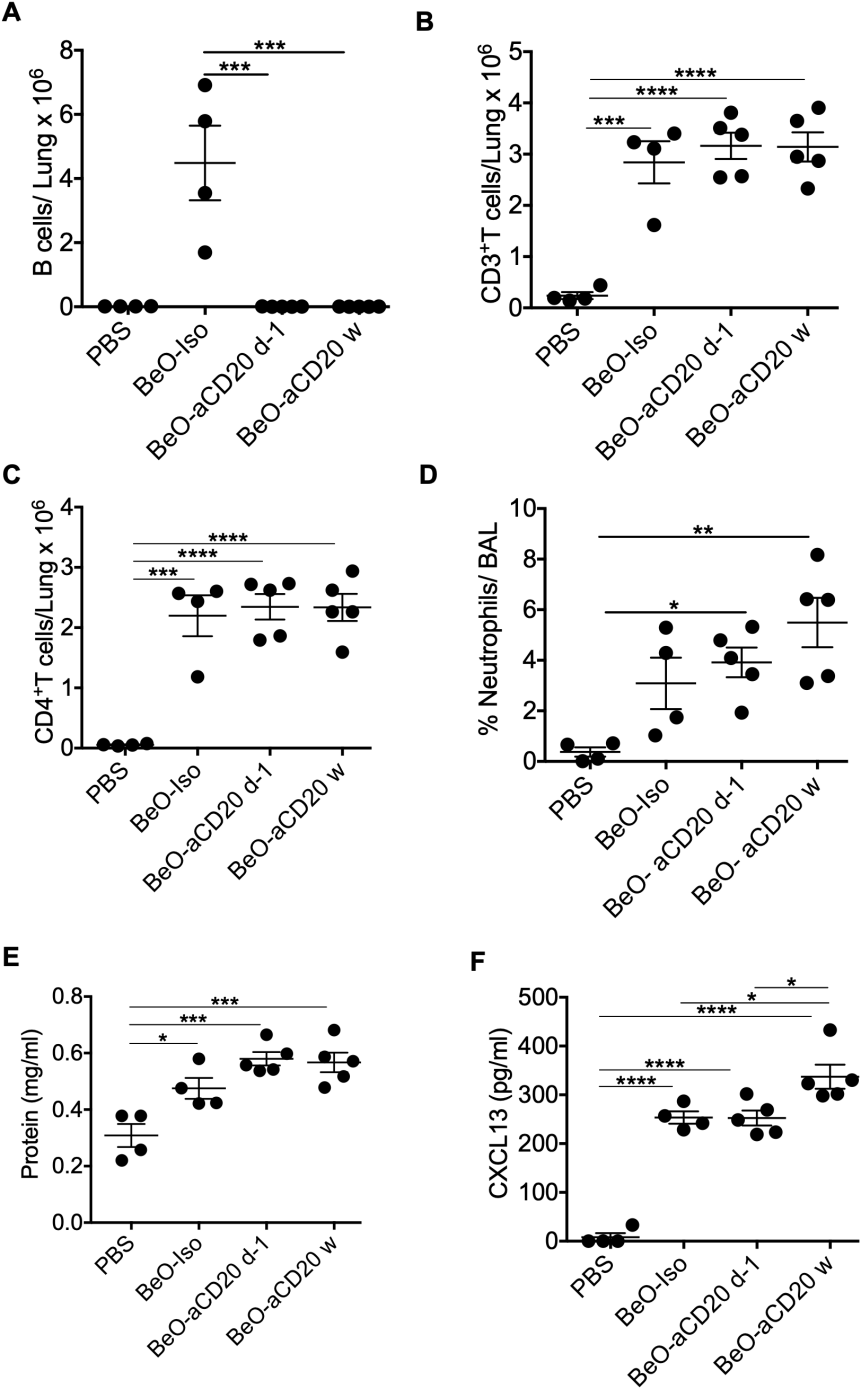
B cells are dispensable for CD4^+^ T cell recruitment and CXCL13 expression. HLA-DP2 FVB/N Tg mice were sensitized with three doses of BeO (100 μg) on days 0, 1, and 2. Mice were then randomly assigned and treated with PBS, isotype control monoclonal antibody (Iso), or an anti-CD20 monoclonal antibody one time (d-1), or weekly (w). **(A)** Total B cells **(B)** CD3^+^ T cells, and **(C)** CD4^+^ T cells in the lungs of BeO-exposed mice on day 21. **(D)** Percent neutrophils were examined in the BAL of BeO-exposed mice. Graph plots show protein leak **(E)** and CXCL13 **(F)** in the bronchoalveolar lavage fluid (BALF) of BeO exposed mice. Data are representative of two independent experiments, four mice per group. One-way ANOVA was used to test statistical differences among the groups. P<0.05 (*) is considered statistically significant. P < 0.05 (*), P < 0.01 (**), P < 0.001 (***), P < 0.0001 (****).

BeO exposure leads to the recruitment of both B cells and CD4^+^ T cells to the lungs of FVB/N-DP2 Tg mice. We decided to investigate if B cell depletion would impact the recruitment of CD4^+^ T cells and the presence of CXCL13 in the lungs. B cells were depleted using an anti-CD20-mAb (200 μg) at day -1 or weekly (w). B cell depletion was confirmed in the lungs and spleen on day 21 (**Supplementary Figure 5**). Treatment with an anti-CD20 mAb one time or weekly resulted in a significant decrease in B cell recruitment to the lungs of FVB/N-DP2-Tg mice exposed to BeO using the standard sensitization/boost protocol (**Figure 6A**). In mice treated with isotype or anti-CD20 mAb, we found no differences in the frequency of recruited total CD3^+^ T cells and CD4^+^ T cells in the presence or absence of B cells (**Figures 6B, C**). Next, we assessed lung injury in the absence of B cells by examining neutrophil recruitment and protein release in response to BeO exposure in the BALF. Both neutrophils and protein leakage showed a trend suggesting increased inflammation with the loss of B cells (**Figures 6D, E**). Interestingly, the levels of CXCL13 were significantly different among the groups (6F). Overall, our data suggests that B cells are not required for the recruitment of CD4^+^ T cells to the lungs.

### CD4^+^ T cells control optimal B cell recruitment to the lung in BeO-exposed HLA-DP2 Tg mice

**Figure 7:**
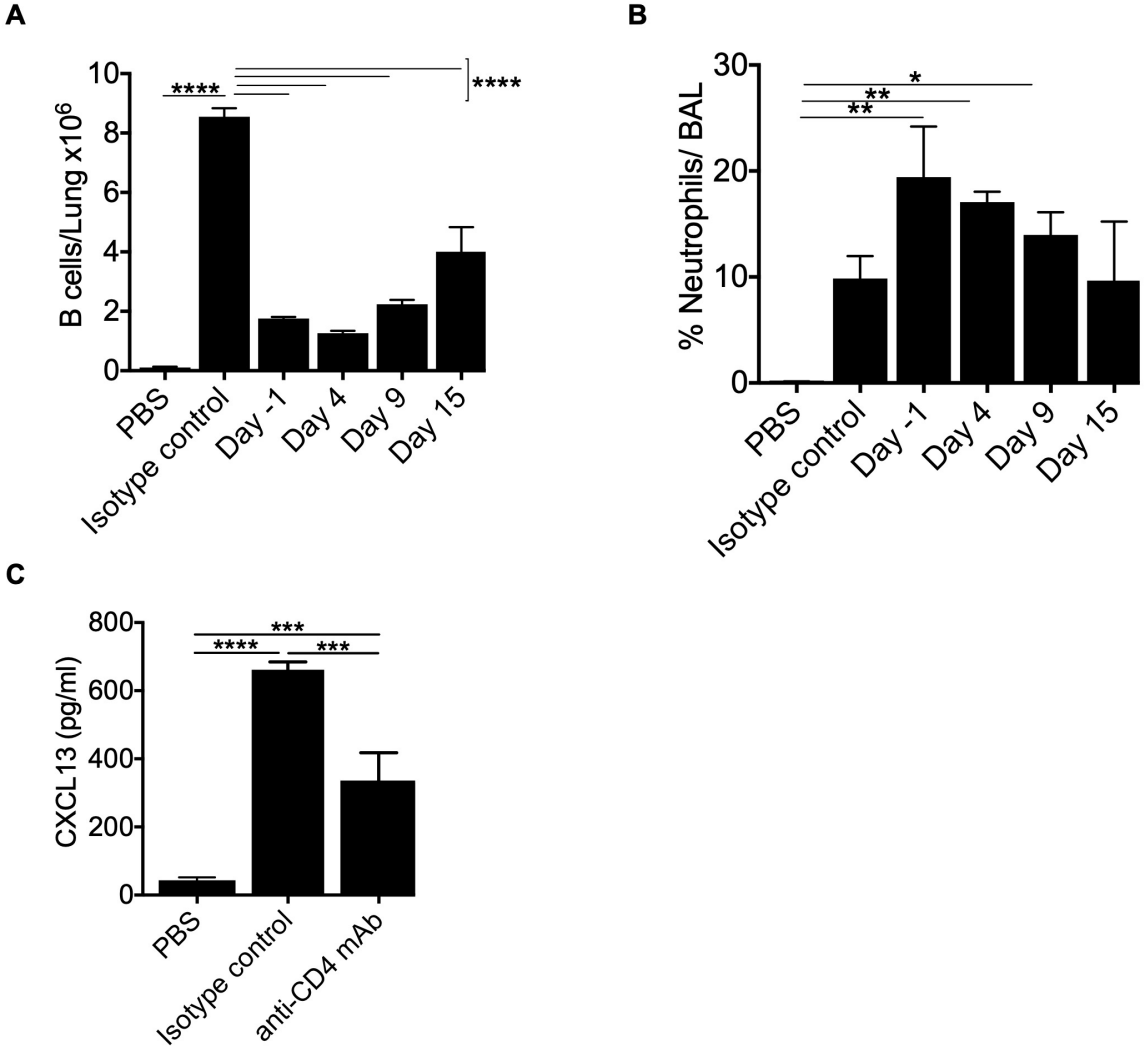
CD4^+^ T cells control optimal B cell recruitment to the lung in BeO-exposed HLA-DP2 Tg mice. HLA-DP2 FVB/N Tg mice were sensitized with BeO on days 0, 1, and 2. Mice were treated with either PBS, an isotype control antibody, or an anti-CD4 monoclonal antibody (100 μg) one day before the experiment (d-1), day 4, day 9, or day 15. **(A)** Total B cells in the lungs on day 21. **(B)** Percent neutrophils in the BAL on day 21. **(C)** CXCL13 levels were examined in the BALF of BeO-exposed HLA-DP2-Tg mice on day 21, treated with either PBS, an isotype control antibody, or an anti-CD4 monoclonal antibody (100 μg) one day before the experiment. Data are representative of two independent experiments, four mice per group. One-way ANOVA was used to test statistical differences among the groups. P < 0.05 (*) is considered statistically significant. P < 0.05 (*), P < 0.01 (**), P < 0.001 (***), P < 0.0001 (****).

Our leukocyte migration data indicated a temporal correlation between CD4^+^ T cells and B cell recruitment to the lungs. To examine the role of CD4^+^ T cells in B cell recruitment, we depleted CD4^+^ T cells in HLA-DP2 Tg FVB/N mice at day -1 (before the start of treatment with three doses of BeO (100 μg), day 4 (two days post sensitization), day 9 (seven days post sensitization), or day 15 (13 days post sensitization) using an anti-CD4 monoclonal antibody. To confirm CD4^+^ T cell depletion, blood was collected from an isotype control antibody or anti-CD4 monoclonal antibody-treated mice on day 3. Mice treated with anti-CD4 monoclonal antibody showed a complete absence of CD4 T cells in the blood (**Supplementary Figure 6**). CD4^+^ T cell depletion at various time points impacted overall B cell recruitment to the lungs of BeO-exposed mice. Mice depleted of CD4^+^ T cells on day 15 showed a slight increase in the number of B cells in the lungs suggesting that the B cells observed in the lungs are recruited with the help of CD4^+^ T cells, and late depletion moderately affected the presence of B cells in the lungs (**Figure 7A**). Additionally, early depletion of CD4^+^ T cells resulted in increased inflammation, evidenced by the increase in neutrophils in the BALF; however, the depletion of CD4^+^ T cells at day 15 did not lead to significant differences in the neutrophil percentage compared to the isotype-treated mice (**Figure 7B**). We also examined the effect of CD4^+^ T cell depletion on the levels of CXCL13 in the lungs of BeO exposed mice. HLA-DP2 Tg mice treated with anti-CD4 mAb showed a significant reduction in CXCL13 compared to the isotype-treated group (**Figure 7C**). Altogether, this data suggests that CD4^+^ T cells are critical for B cell recruitment and B cell-mediated protection against BeO-induced lung injury, and it is required to maintain the levels of CXCL13 in the lungs.

### BeO exposure induces activation of tissue-specific B cells

**Figure 8:**
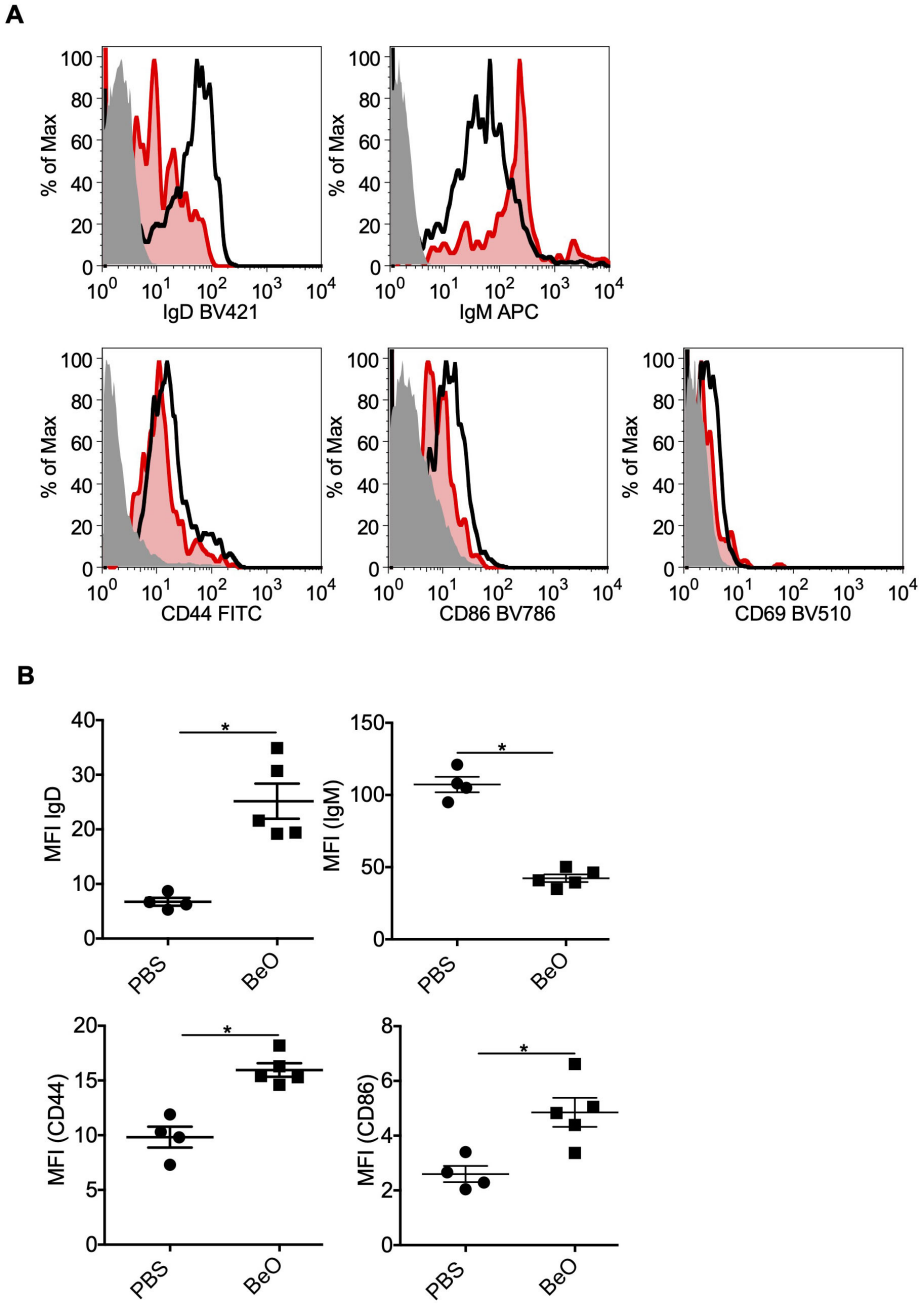
Phenotyping of tissue-specific B cells in the lungs of BeO-exposed HLA-DP2 Tg mice. Mice were sensitized and boosted with BeO (100 μg) on days 0, 1, 2, 14, 15, 18, and 19. **(A)** Lung B cell-surface expression of IgD (top left), IgM (top right), CD44 (bottom left), and CD86 (bottom right) were examined on day 21. The histogram represents FMO control (solid-grey), PBS (red), and BeO (black) groups. **(B)** Graph plots show the mean fluorescence intensity (MFI) of various molecules on the tissue-specific B cells. Data are representative of two independent experiments containing n=3-5 mice/group. Significance was determined by the Mann-Whitney t-test. P<0.05 (*) is considered statistically significant.

We previously showed that B cells in the lungs are activated based on the increased expression of costimulatory molecules such as CD40 and CD86 (20). To further characterize the phenotypes of B cells in the lungs of mice exposed to BeO, we have examined the expression of IgM and IgD by flow cytometry. We found that in BeO-exposed mice, there was an approximately 3-fold increase in the MFI of IgD compared to PBS-exposed mice (**Figures 8A, B**). Interestingly, we observed a decreased expression of IgM suggesting exposure to cognate antigen and B cell activation. To determine the activation status of the B cells, we examined the expression of CD44 and CD86 and found that BeO exposure leads to increased expression of these molecules (**Figures 8A, B**). CD44 is also described as a marker for tissue-specific B cells (30).

### RNA-sequencing of lung tissue-specific B cells from BeO-exposed HLA-DP2 Tg mice implicates genes involved in interferon signaling, tight junction integrity, and antigen presentation

**Figure 9:**
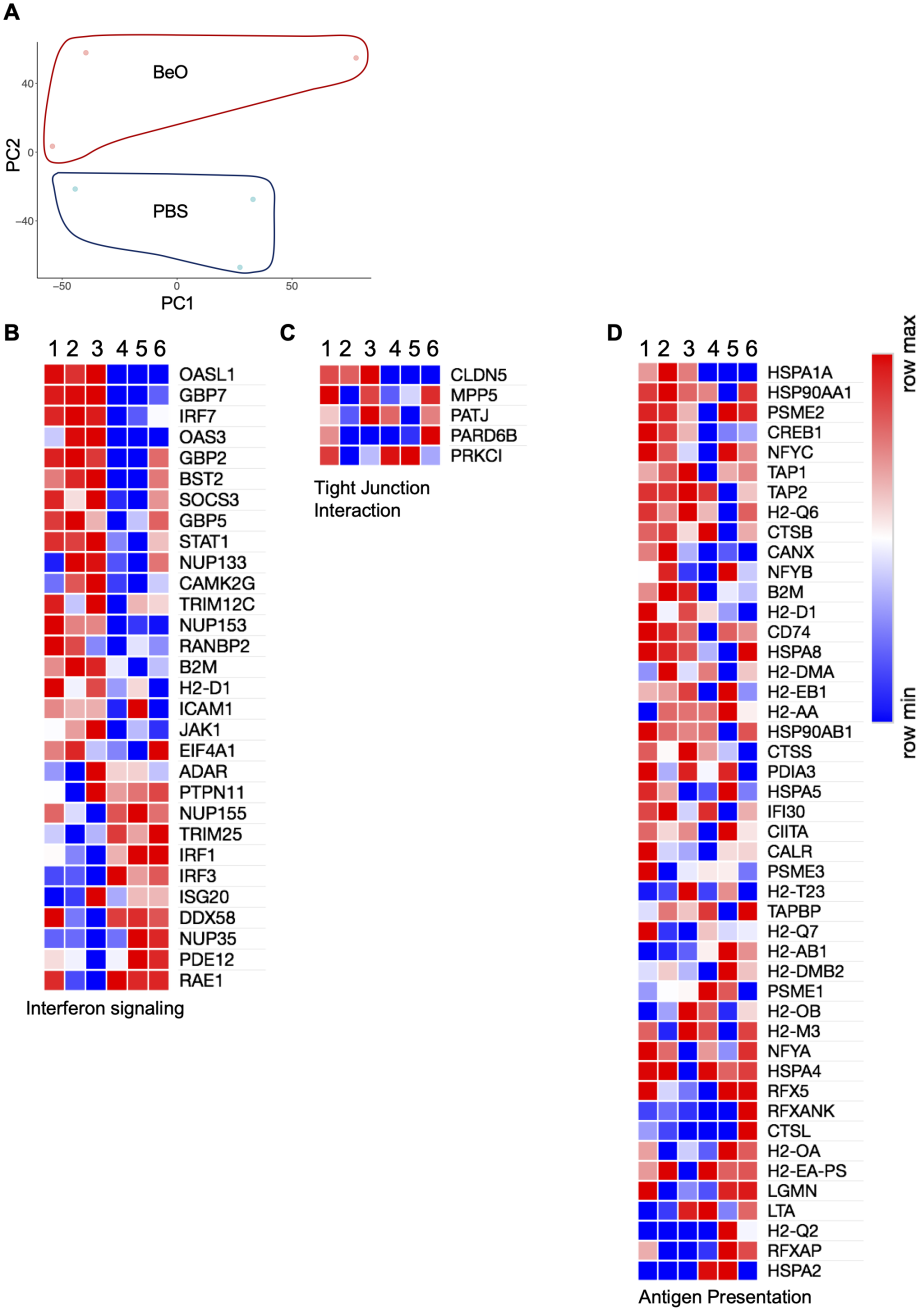
RNA-sequencing of lung tissue-specific B cells from BeO exposed HLA-DP2 Tg mice identify genes involved in increased activation and antigen presentation. HLA-DP2 FVB/N Tg mice were sensitized and boosted with BeO on days 0, 1, 2, 14, 15, 18, and 19. On day 21, IVCD45- B cells were sorted by FACS. B cells were pooled from n=5 mice/per experiment, and RNA samples (1, 2, 3) were prepared. The numbers (1, 2, 3) represent individual experiments. RNA sequencing was performed and compared against control samples (4, 5, 6), prepared from CD23^+^ follicular B cells sorted from the spleens of WT mice. **(A)** Principal component analysis showing the distribution of control and BeO BeO-exposed samples. Differentially expressed genes are associated with interferon signaling **(B)**, tight junction interactions **(C)**, and antigen presentation **(D)**.

BeO exposure induces robust recruitment of B cells to the lungs of HLA-DP2 Tg mice. These B cells were shown to be protective in CBD (20), however, the cellular and molecular profile of the recruited B cells has not been previously described. To examine these changes, we performed RNA-sequencing on the tissue-specific B cells sorted from BeO-exposed lungs and PBS-treated spleen. The CD23^+^ B cells were sorted from the spleen of control mice because the frequency of tissue-specific B cells is almost negligible in the lungs of PBS-treated mice (21). Principal component analysis (PCA) plots showed B cells from BeO-exposed lungs and CD23^+^ PBS-treated spleen aligned separately (**Figure 9A**). Next, we performed Gene Ontology (GO)-pathway analysis using upregulated or downregulated genes between lung B cells and splenic control B cells. In BeO-treated mice, tissue-specific lung B cells (1, 2, 3) showed enhanced expression of genes (*OASL1, OAS3, STAT1*) involved in suppressing interferon-induced inflammation (34, 35), promoting class switch recombination and generation of memory B cells (36) (**Figure 9B**). Additionally, we observed high expression of *CLDN5* in B cells (**Figure 9C**), suggesting an important role of B cells in maintaining the cellular aggregates in the lungs of BeO-exposed mice. B cells are professional antigen-presenting cells (29), which allow them to present the antigens complexed on the MHC molecules to both CD4^+^ and CD8^+^ T cells. RNA sequencing of tissue-specific B cells shows increased expression of genes involved in antigen presentation (**Figure 9D**). Overall, our RNA sequencing data showed changes in the transcriptional profile of tissue-specific B cells, suggesting their role in mediating suppression and aggregate formation.

## Discussion

The occupational hazards associated with metals and their derivatives are a major health concern. Here we focused on studying the effect of Beryllium oxide (BeO), a metal derivative heavily used in aviation, automobiles, ceramics, and sporting industries on the recruitment and transcriptional profile of tissue-specific B cells. Previously, using a humanized model of chronic beryllium disease, we demonstrated a protective role of tissue-resident regulatory CD4^+^ T cells and B cells in CBD (18, 19, 30). However, how these adaptive immune cells traffic to the lungs after BeO exposure was previously not well understood. Using a multiprong approach, we expanded on the roles of interleukin signaling, chemokine-chemokine receptor signaling, and CD4^+^ T cells and found that all 3 factors play an important role in driving B cell recruitment to the lungs.

Interleukins are important molecules that are released during sterile, and pathogen-induced inflammation. Among the interleukins, the IL-1 family of cytokines and receptors is involved in regulating immunological responses which includes the development, activation, and migration of immune cells (31). IL-1α and IL-1β are secreted in the alveolar space in response to BeO exposure in mice (18). IL-1α is constitutively expressed in epithelial and mesenchymal cell types of healthy subjects while IL-1β is primarily induced under pathologic conditions. IL-1α is also an “alarmin” released from injured or dying cells resulting in the secretion of chemokine molecules which then recruit the immune cells to the site of injury. We found that in BeO-exposed mice, IL-1α and IL-1β require IL-1R1 signaling to recruit adaptive immune cells to the lungs.

Administration of neutralizing antibodies specific to IL-1α was shown to reduce inflammation in cigarette smoke (CS)-exposed and H1N1-infected mice (37). In SAMP1/YitFc mice which spontaneously develop Crohn’s-like ileitis, administration of an anti-IL-1α antibody protects against inflammation and damage. Additionally, anti-IL-1α treatment ameliorated chronic ileitis and protected mice from developing acute DSS-induced colitis (38). Anti-IL-1α treatment in our CBD mouse model demonstrated a significant reduction in B cell recruitment (**Figure 2**). However, the anti-IL-1α treatment also prevented the recruitment of CD3^+^ T cells and Be-induced inflammation in the lungs indicated by a reduction in neutrophil recruitment. This reduced response suggests that IL-1α is a part of the initiating molecular complex that controls both innate and adaptive immune responses. Similar responses were seen with a recombinant version of the IL-1 receptor antagonist, Anakinra, where the blocking of IL-1R1 prevented the recruitment of adaptive immune cells including B cells to the lungs. Mice exposed to beryllium and treated with Anakinra showed reduced lung injury. Anakinra therapy was shown to be beneficial in reducing inflammation in COVID-19 (35) and in the mouse model of acute lung injury (ALI) that is commonly used to study acute respiratory distress syndrome (ARDS) (36).

Chemokines are small molecules that regulate directional leukocyte migration and activation during inflammatory and homeostatic processes in a time- and site-dependent manner (39, 40). CXCL13 is constitutively expressed in organs regulating the trafficking of adaptive immune cells. CXCL13 is the only ligand for CXCR5, which is expressed on both B and T cells. Increased CXCL13 production is reported in the lungs of BeO-treated mice. We showed that loss of B cells resulted in an increased level of CXCL13 in the BAL fluid of mice treated with BeO. The increasing trend observed for CXCL13 levels in the BALF of B cell-depleted mice may be due to the increased presence of CD4^+^ T cells that direct the secretion of CXCL13 from immune and non-immune cells and the absence of B cells utilizing it through the expression of CXCL13 receptor, CXCR5 (**Figure 6**). In the CXCR5-deficient mice, both the frequency and number of B cells and T cells in the alveolar space were greatly reduced suggesting its importance in the recruitment of immune cells to the lungs of BeO exposed mice in a CXCL13 dependent fashion (41).

Our CD4^+^ T cell depletion and kinetics studies revealed an important role for CD4^+^ T cells in recruiting B cells to the alveolar space of BeO-exposed mice and maintaining the levels of CXCL13. Time-kinetic experiments established a protective role for B cells in CBD, as indicated by reduced neutrophils and increased B cell migration. B cells do not function as antigen-presenting cells in CBD (20), in contrast to type 1 diabetes, where B cells serve as critical antigen-presenting cells for the initiation of T cell-mediated autoimmune diabetes in non-obese diabetic mice (42). In the model of autoimmune diabetes, treatment with an anti-CD20 mAb prevents and reverses diabetes in mice (43). The increased T cells, neutrophils, and protein leak in the alveolar space of mice depleted with an anti-CD20 mAb suggest a suppressive role for B cells in CBD (**Figure 6**).

Recently, multiple studies have described the role of tissue-resident B cells in various diseases, including influenza virus infection (27). The intravenous injection with fluorescently labeled anti-CD45 (IV CD45) monoclonal antibody (mAb) allows tissue cells to be differentiated from those in circulation (27–29). Using this methodology, we have identified tissue-specific B cells in the lungs of BeO-exposed mice. These B cells have reduced surface IgM and increased IgD expression. An elegant study by Noviski et al. demonstrated that IgM but not IgD is downregulated in autoreactive B cells, and this observation is attributed to the affinity of IgM and IgD B cell receptors towards endogenous antigens (44). Based on this observation, we believe follicular B cells in BeO-exposed mice might respond to endogenous antigens exposed due to tissue damage, resulting in the downregulation of IgM BCRs. However, based on the findings of Choi et al., it is also possible that a non-conventional class switch recombination might have occurred in BeO exposed mice, causing IgD class switch recombination (45). Mice and humans exposed to viruses and bacteria showed upregulation of CD44, CD62L, CXCR3, CCR6, and CD69 on tissue-resident B cells (29, 30, 46, 47). Our findings also confirmed that B cells in the tissues were activated, as indicated by increased expression of CD44 and CD86. The expression of CD44 suggests that these B cells could be tissue-resident B cells (30). Unlike others, in our studies, CD69 staining on IV CD45- B cells in the lungs was absent (**Figure 8**). Our RNA-sequencing data on the lung tissue-specific B cells showed an upregulation of genes involved in tight junction formation. Additional experiments will be required to further elucidate the precise mechanisms B cells use to suppress Be-induced inflammation.

In summary, our findings show that IL-1/IL-1R1 signaling and CD4^+^ T cells play an important role in controlling the recruitment of B cells to the lungs in response to BeO exposure. The recruitment of B cells was found to be dependent on the CXCL13/CXCR5 signaling. Further, the introduction of B cells to the lungs of BeO-exposed μMT mice confirms the role of B cells in protection against lung injury. Taken together, our work begins to elucidate the mechanisms involved in B cell recruitment in response to sterile particulate exposure and contributes to the existing knowledge of B cell-mediated protection in Be-induced lung injury. It also lays the groundwork for developing therapies for treating CBD and similar lung diseases by targeting innate immune pathways in the early phase of the disease.

## Data Availability

The data presented in the study are deposited in the Gene Expression Omnibus, accession number GSE280133.

## Supplementary material

Supplementary Figure 1 Gating strategy used for the examination of IVCD45-lymphocytes. WT or HLA-DP2 Tg mice were exposed to PBS or BeO. On day 12 or day 21, mice were intravenously injected with anti-mouse CD45 mAb antibody (clone 30-F11), 2 minutes before sacrifice. Lymphocytes were examined in the tissue by flow cytometry. Cells in circulation were identified by positive labeling with CD45. Of note, multiple fluorochromes were used for the identification of CD3^+^ and B220/CD19^+^ cells as can be found throughout the text.

Supplementary Figure 2 Frequency of B cells in the spleen. Mice were exposed to PBS or BeO on days 0, 1, 2, 14, 15, 18, and 19. On day 21, B cells were examined in the spleen. **(A)** Dot plots and **(B)** frequency of B cells in PBS or BeO-treated WT or IL-1R1^-/-^ mice. Data are representative of three independent experiments having 3-5 mice per group. One-way ANOVA was used to test statistical differences among the groups. P<0.05 (*) is considered statistically significant.

Supplementary Figure 3 Frequency of B cells in the alveolar space of BeO exposed mice. WT and μMT mice were exposed to PBS or BeO on days 0, 1, 2, 14, 15, 18, and 19. On day 21, B cells were examined in the lungs. **(A)** Frequency and **(B)** Number of B cells in BeO-treated WT or μMT mice. Representative data (Mean +/- SEM) from two experiments (3-7 mice per group) is shown. Significance was determined by the Mann-Whitney t-test. P<0.05 (*) is considered statistically significant.

Supplementary Figure 4 Frequency of B cells in the spleen. Mice were exposed to PBS or BeO on days 0, 1, 2, 14,1 5, 18, and 19. On day 21, B cells were examined in the spleen. **(A)** Dot plots and **(B)** Frequency of B cells in PBS or BEO treated WT or CXCR5^-/-^ mice. Data are representative of three independent experiments having 3-5 mice per group. One-way ANOVA was used to test statistical differences among the groups. P<0.05 (*) is considered statistically significant.

Supplementary Figure 5 B cell depletion examined in the lungs and spleen of BeO-exposed mice. HLA-DP2 FVB/N Tg mice were treated with isotype control monoclonal antibody (Isotype), or an anti-CD20 monoclonal antibody on (d-1) and sensitized with 3 doses of BeO (100 μg) on days 0, 1, and 2. Frequency of B cells in the lungs **(A, B)** and spleen **(C, D)** examined on day 21 in isotype or anti-CD20 treated groups. Data are representative of three independent experiments having 3-5 mice per group. Significance was determined by the Mann-Whitney t-test. P<0.05 (*) is considered statistically significant.

Supplementary Figure 6 CD4 T cell depletion examined in the blood. HLA-DP2 FVB/N Tg mice were treated with either an isotype control antibody or an anti-CD4 monoclonal antibody (100 μg) one day before the experiment. Flow plots **(A)** and cumulative graph plots **(B)** show the frequency of CD4^+^ T cells examined in the blood on day 3 post-treatment. Data are representative of three independent experiments having 3-5 mice per group. Significance was determined by the Mann-Whitney t-test. P<0.05 (*) is considered statistically significant.
