# Supplementary figures and images for: Interleukin-1 signaling and CD4+ T cells control B cell recruitment to the lungs in chronic beryllium disease
Source: Front Immunol. 2025 Jan 28;16:1479348. doi: 10.3389/fimmu.2025.1479348 (PMC11810750; doi:10.3389/fimmu.2025.1479348)

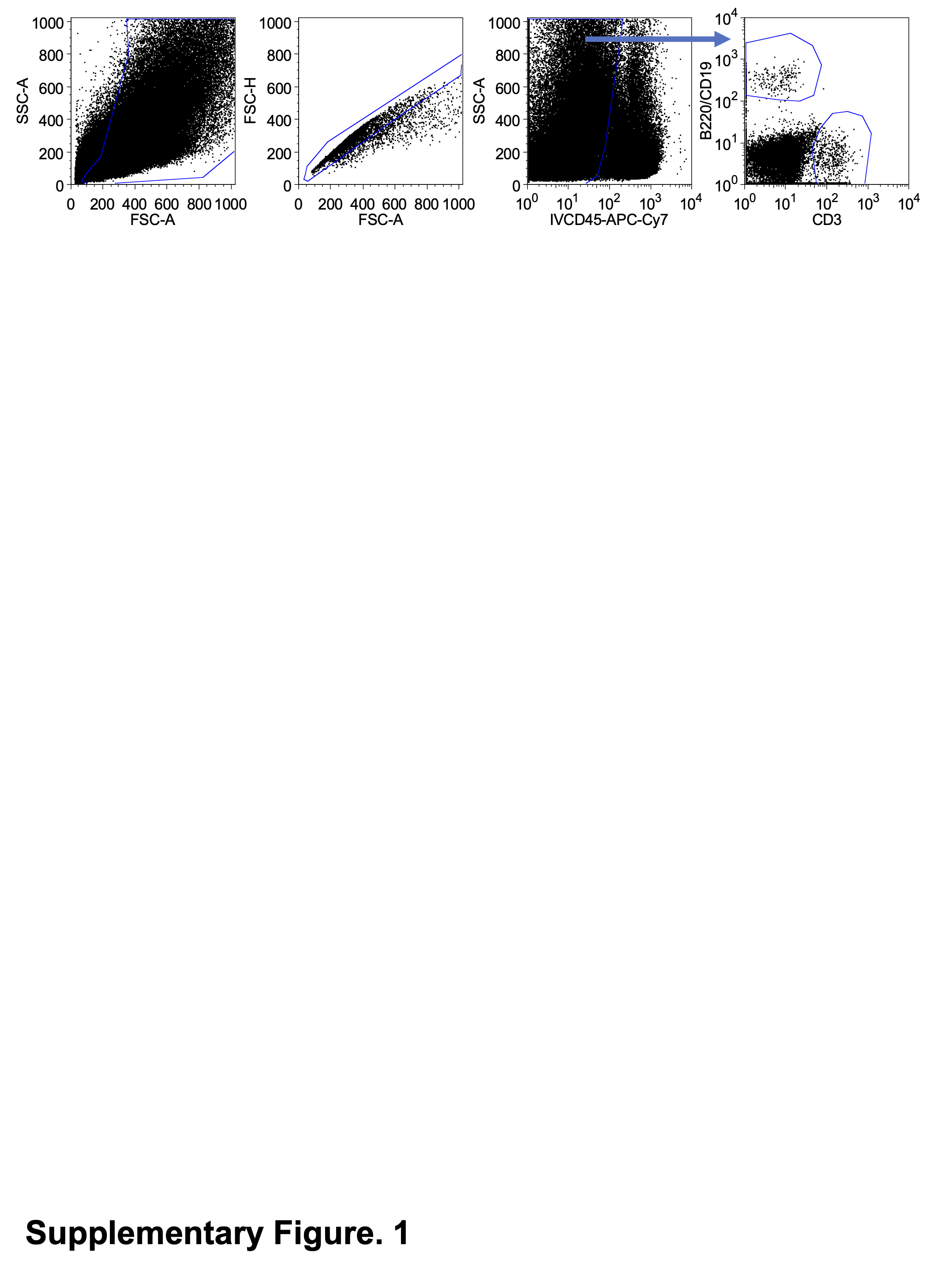

Supplement: Supplementary Figure 1 — Gating strategy used for the examination of IVCD45-lymphocytes. WT or HLA-DP2 Tg mice were exposed to PBS or BeO. On day 12 or day 21, mice were intravenously injected with anti-mouse CD45 mAb antibody (clone 30-F11), 2 minutes before sacrifice. Lymphocytes were examined in the tissue by flow cytometry. Cells in circulation were identified by positive labeling with CD45. Of note, multiple fluorochromes were used for the identification of CD3+ and B220/CD19+ cells as can be found throughout the text. [file Image1.tiff]

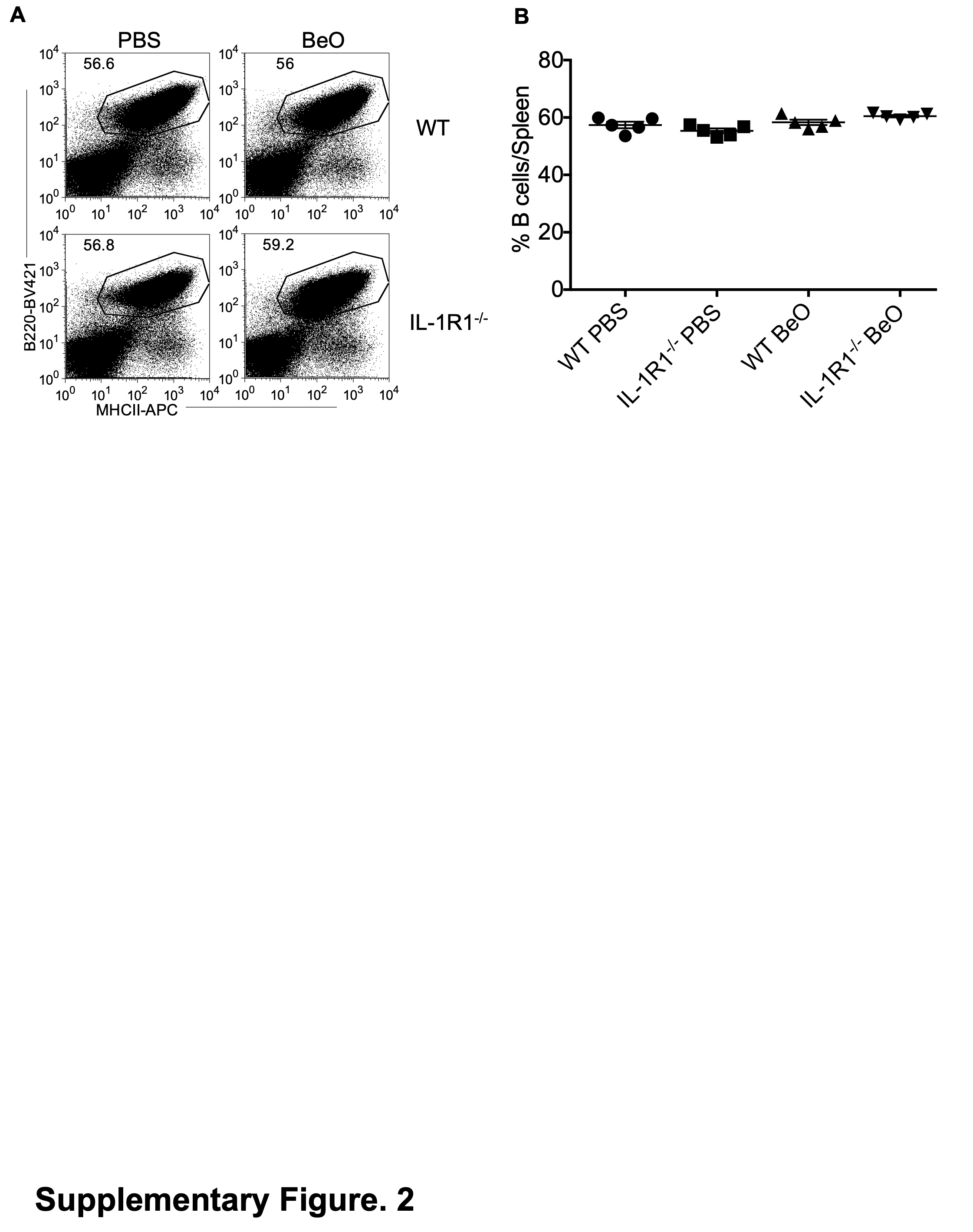

Supplement: Supplementary Figure 2 — Frequency of B cells in the spleen. Mice were exposed to PBS or BeO on days 0, 1, 2, 14, 15, 18, and 19. On day 21, B cells were examined in the spleen. (A) Dot plots and (B) frequency of B cells in PBS or BeO-treated WT or IL-1R1-/- mice. Data are representative of three independent experiments having 3-5 mice per group. One-way ANOVA was used to test statistical differences among the groups. P<0.05 (*) is considered statistically significant. [file Image2.tiff]

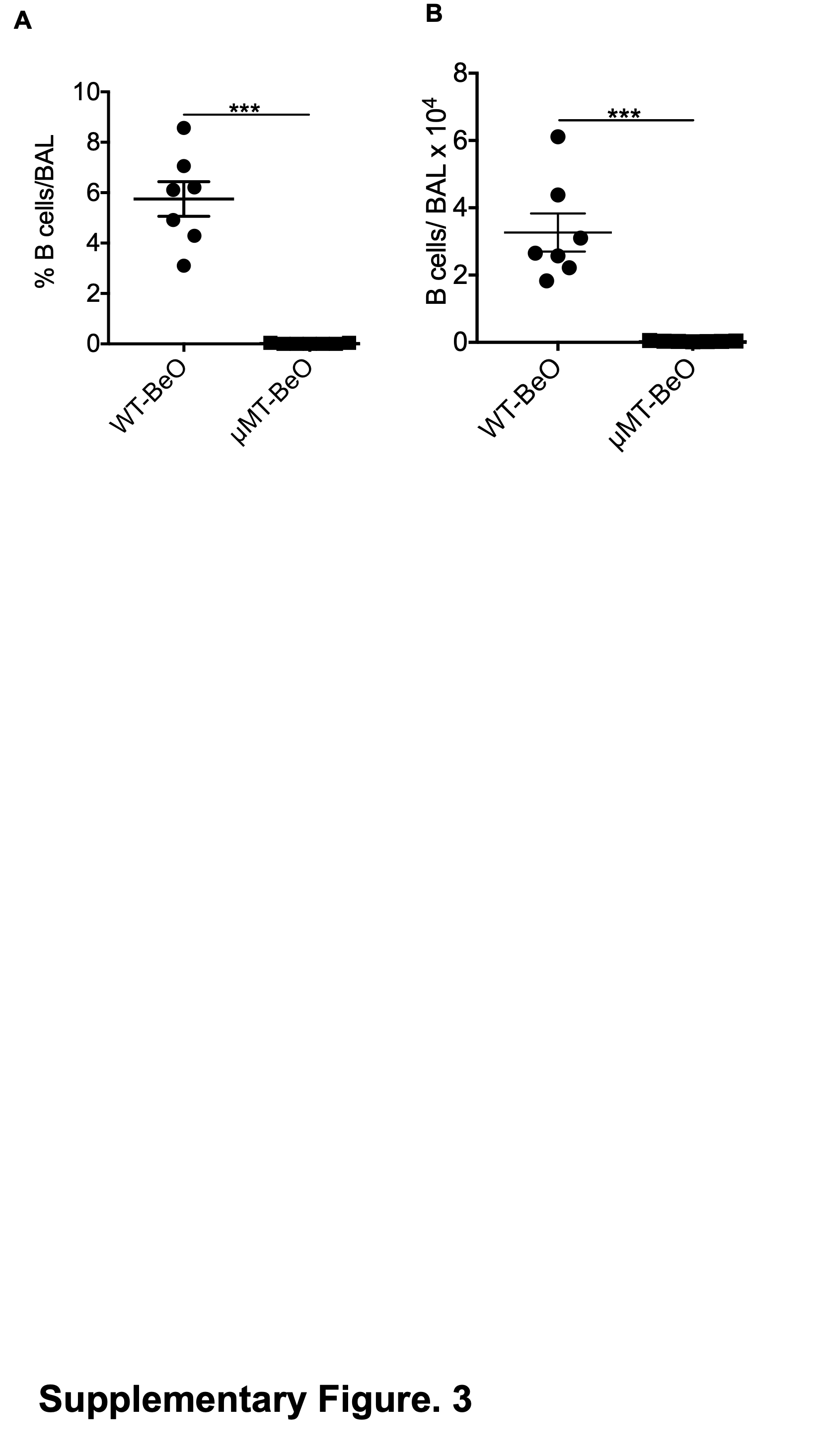

Supplement: Supplementary Figure 3 — Frequency of B cells in the alveolar space of BeO exposed mice. WT and μMT mice were exposed to PBS or BeO on days 0, 1, 2, 14, 15, 18, and 19. On day 21, B cells were examined in the lungs. (A) Frequency and (B) Number of B cells in BeO-treated WT or μMT mice. Representative data (Mean +/- SEM) from two experiments (3-7 mice per group) is shown. Significance was determined by the Mann-Whitney t-test. P<0.05 (*) is considered statistically significant. [file Image3.tiff]

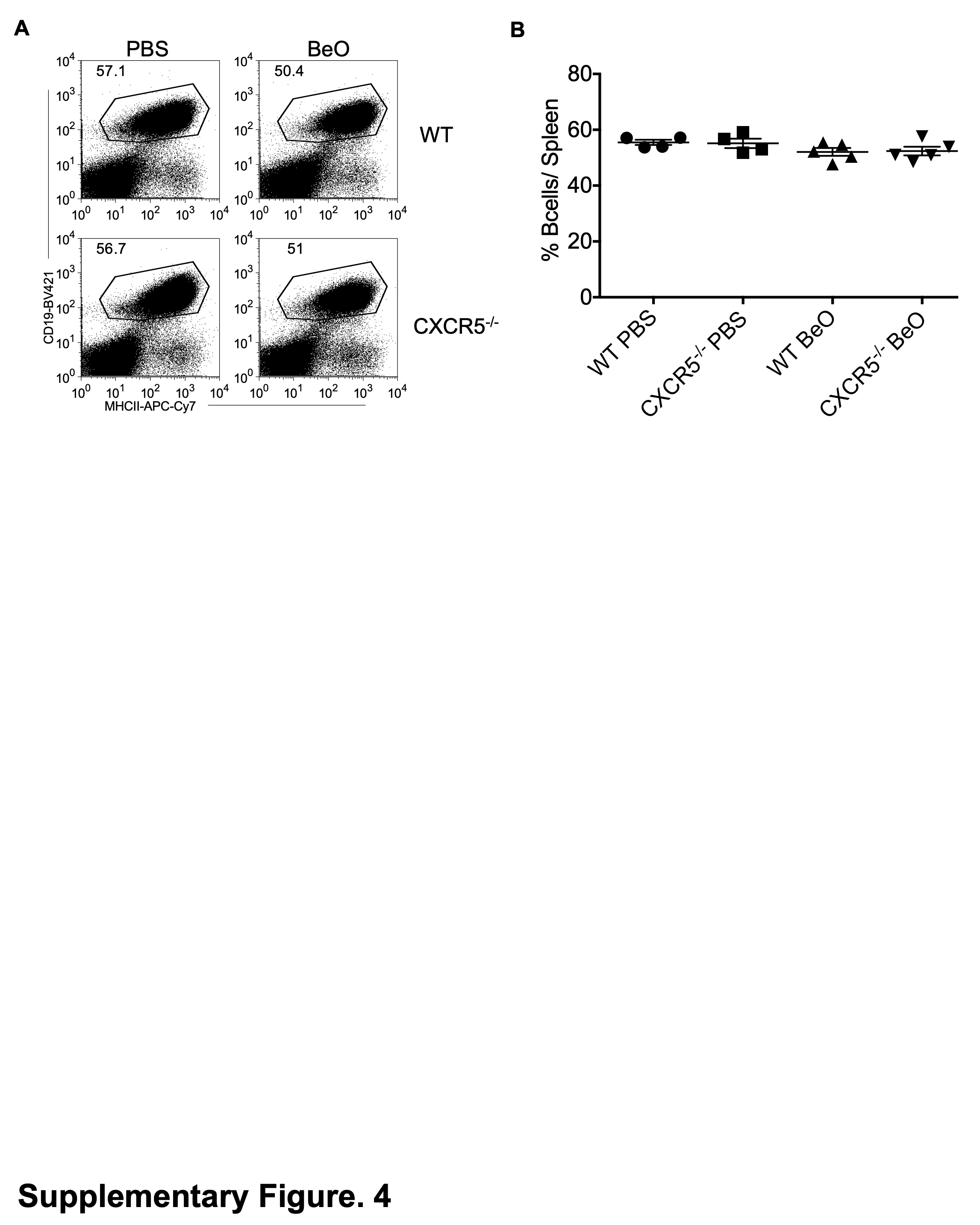

Supplement: Supplementary Figure 4 — Frequency of B cells in the spleen. Mice were exposed to PBS or BeO on days 0, 1, 2, 14,1 5, 18, and 19. On day 21, B cells were examined in the spleen. (A) Dot plots and (B) Frequency of B cells in PBS or BEO treated WT or CXCR5-/- mice. Data are representative of three independent experiments having 3-5 mice per group. One-way ANOVA was used to test statistical differences among the groups. P<0.05 (*) is considered statistically significant. [file Image4.tiff]

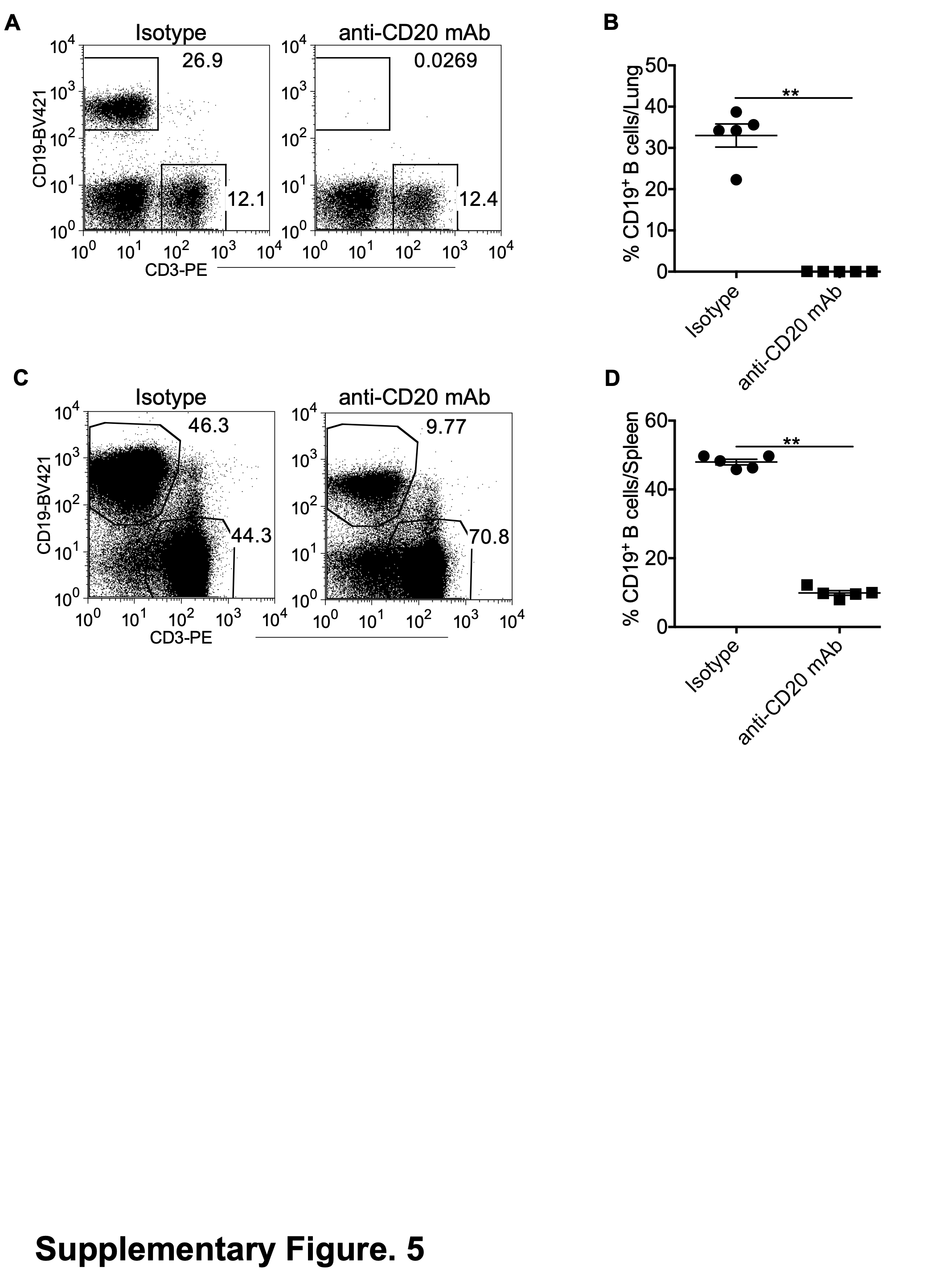

Supplement: Supplementary Figure 5 — B cell depletion examined in the lungs and spleen of BeO-exposed mice. HLA-DP2 FVB/N Tg mice were treated with isotype control monoclonal antibody (Isotype), or an anti-CD20 monoclonal antibody on (d-1) and sensitized with 3 doses of BeO (100 μg) on days 0, 1, and 2. Frequency of B cells in the lungs (A, B) and spleen (C, D) examined on day 21 in isotype or anti-CD20 treated groups. Data are representative of three independent experiments having 3-5 mice per group. Significance was determined by the Mann-Whitney t-test. P<0.05 (*) is considered statistically significant. [file Image5.tiff]

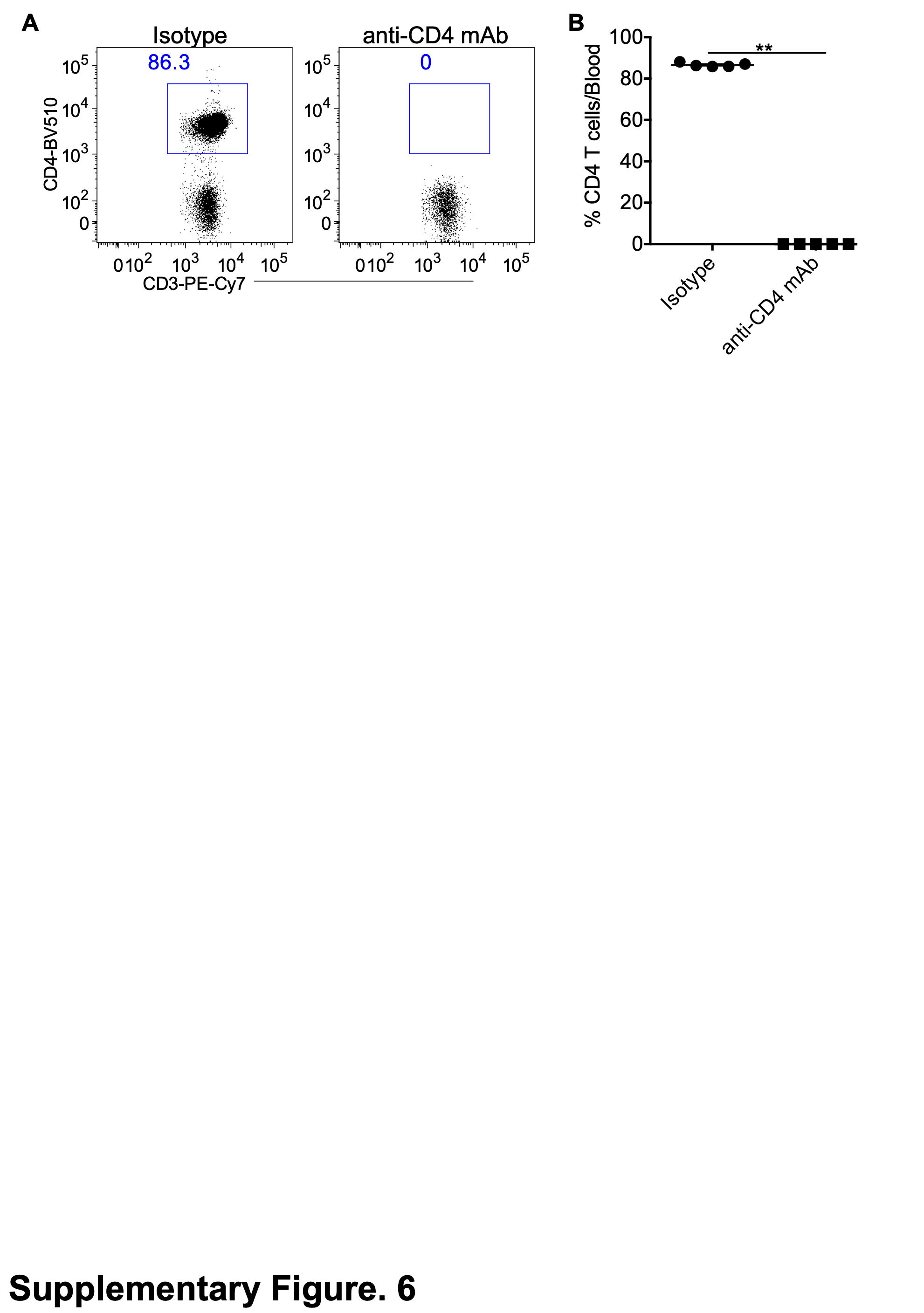

Supplement: Supplementary Figure 6 — CD4 T cell depletion examined in the blood. HLA-DP2 FVB/N Tg mice were treated with either an isotype control antibody or an anti-CD4 monoclonal antibody (100 μg) one day before the experiment. Flow plots (A) and cumulative graph plots (B) show the frequency of CD4+ T cells examined in the blood on day 3 post-treatment. Data are representative of three independent experiments having 3-5 mice per group. Significance was determined by the Mann-Whitney t-test. P<0.05 (*) is considered statistically significant. [file Image6.tiff]
